# Investigation of Structural Mimetics of Natural Phosphate Ion Binding Motifs

**DOI:** 10.3390/molecules20023354

**Published:** 2015-02-16

**Authors:** Evgeny A. Kataev, Tatiana A. Shumilova

**Affiliations:** Institute of Chemistry, Faculty of Natural Sciences, Technische Universität Chemnitz, 09107 Chemnitz, Germany; E-Mail: sh.tat.93@gmail.com

**Keywords:** phosphate binding, peptide mimetics, macrocycle, glycine-rich loop, synthetic receptor, host-guest chemistry, anion binding

## Abstract

Phosphates are ubiquitous in biology and nearly half of all proteins interact with their partners by means of recognition of phosphate residues. Therefore, a better understanding of the phosphate ion binding by peptidic structures is highly desirable. Two new receptors have been designed and synthesized and their anion binding properties in an acetonitrile solution have been determined. The structure of hosts mimics a part of the kinase active site that is responsible for the recognition of the phosphate residue. New hosts contain additional free amino groups with the aim to facilitate coordination of protonated anions, such as dihydrogen phosphate. According to spectrophotometric measurements, stepwise 1:1 and 1:2 binding modes have been observed for both receptors in the presence of acetate, hydrogen sulfate and dihydrogen phosphate. Compared with the acyclic receptor, the macrocyclic receptor has demonstrated a remarkably enhanced selectivity for dihydrogen phosphate over other anions. Fluorometric measurements have revealed different responses of the acyclic and macrocyclic receptors towards anions. However, in both cases, a 5–8 nm hypsochromic shift of fluorescence maximum has been observed upon interaction of acetate and dihydrogen phosphate with receptors.

## 1. Introduction

Phosphates are ubiquitous in biology and nearly half of the proteins interact with their partners by means of recognition of phosphate residues [1,2]. They also play a role of integration between different biochemical events ranging from metabolism and biosynthesis to gene regulation, signal transaction, muscle contraction, and antibiotic resistance. The presence or absence of the phosphate residue in a protein is central in regulating transmembrane and intracellular signal transaction pathways using protein kinases (PKs) [3] and protein phosphatases (PPs) [4]. Thus, detection and sensing of natural phosphorylated species have attracts considerable attention among scientists during the last decade [5,6,7,8]. Analysis of the literature [1] on structural aspects of phosphate binding shows that the predominant part of proteins includes a so-called “P loop”, which is essential for the affinity for the phosphate residue and sometimes referred to as a “giant anion hole”. The amino acid sequences of “P-loops” are glycine-rich with several amino acids—X, referring to any alternative amino acid. Interestingly, for proteins, which bind mononucleotides, the characteristic sequence responsible for phosphate recognition is GXXGXGK(S,T) or GXXX, while for dinucleotide binding the sequence GXGXXG is typical. The reason why nature uses glycine is apparently the flexibility of this moiety, namely the ability to bend the sequence in almost perpendicular direction. For example, in a mononucleotide binding case two glycines are in n + 4 and n + 6 positions, thus they shape the binding site into a cycle [9].

A better understanding of the phosphate ion binding by peptidic structures is therefore highly desirable [10]. To the best of our knowledge, there is no precedent in the literature with the investigation of anion binding properties of model compounds containing several glycine residues in the recognition motif. There have been a number of artificial receptors published, which have structures based on cyclic or acyclic peptides [11]. Some of these receptors can bind the phosphate anion [12,13,14], phosphate esters [15], glucose-1-phosphate [16], and even nucleotides [17,18].

In this work, we explore anion-binding properties of two new compounds that structurally mimic the active site of one of the kinases [19]. The new receptors repeat the glycine-rich “P loop” containing the sequence GGL. This sequence was functionalized from both ends with aromatic compounds with the aim to rigidify the overall structure of the receptors and allow one to investigate binding properties with the help of spectrophotometric methods. One of the receptors has a tweezers-like structure with two terminal amino groups and the other is a macrocycle containing only one free amino group. The amino groups were introduced as hydrogen-bond-acceptor groups to facilitate the coordination of anions bearing protons, such as dihydrogen phosphate. The amino groups in the structure of hosts mimic a guanidinium group, which is found in active sites of most natural phosphate binding proteins.

## 2. Results and Discussion

### 2.1. Design and Synthesis

**Figure 1:**
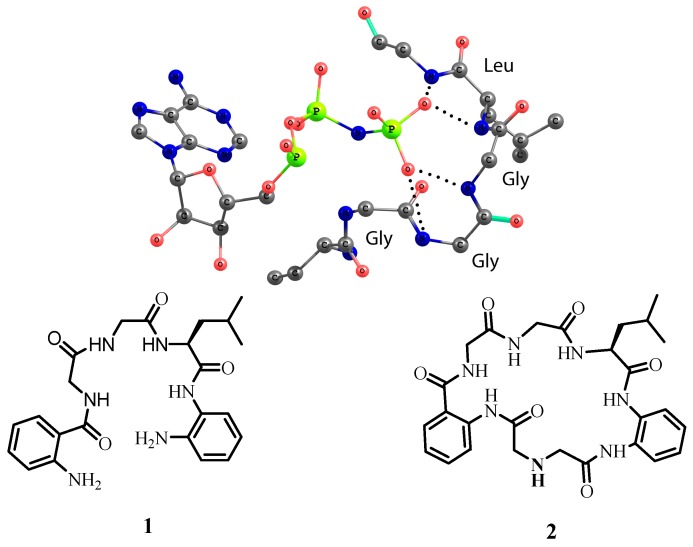
Structure of the active center of 4-diphosphocytidyl-2C-methyl-d-erythritol kinase coordinating AMP-PNP substrate and the structure of model receptors **1** and **2** investigated in this work. Hydrogen bonds are depicted as dotted lines (PDB code 1OJ4).

The design for model receptors relies on the structure of the active center of 4-diphosphocytidyl-2C-methyl-d-erythritol kinase [19]. The amino acid chain forms a loop around the phosphate with GGLG sequence (Figure 1). Interestingly, for the coordination of two oxygen atoms only four NH-amide groups of tripeptide GGL are required. To generate a structural model of this binding motif, we functionalized the GGL sequence with 1,2-phenylenediamine units from the one side and with the anthranilic acid from the other side, respectively. The aromatic rings were introduced with the aim to produce amides from both sides of the sequence and rigidify the overall conformation of receptors. There are two reasons why receptor **1** contains two terminal amino groups. Firstly, amino groups can be protonated and generate additional electrostatic interactions between the receptor and the anion. Secondly, receptor **1** can react with diacids bearing either hydrogen-bond-donor or hydrogen-bond-acceptor groups. In this work, we synthesized macrocyclic receptor **2**, which contains one secondary amino group. Thus, receptors **1** and **2** differ from each other in structure and in number of amino groups available for protonation. For anion binding studies, we used the following anions as their tetrabutylammonium salts: Cl^−^, CH_3_COO^−^, H_2_PO_4_^−^, HSO_4_^−^, and NO_3_^−^. One can expect that interaction of receptors **1** and **2** in organic solvents with hydrogen sulfate and dihydrogen phosphate might be stronger than for other anions because free amino groups can accept the proton from these anions.

**Figure 2:**
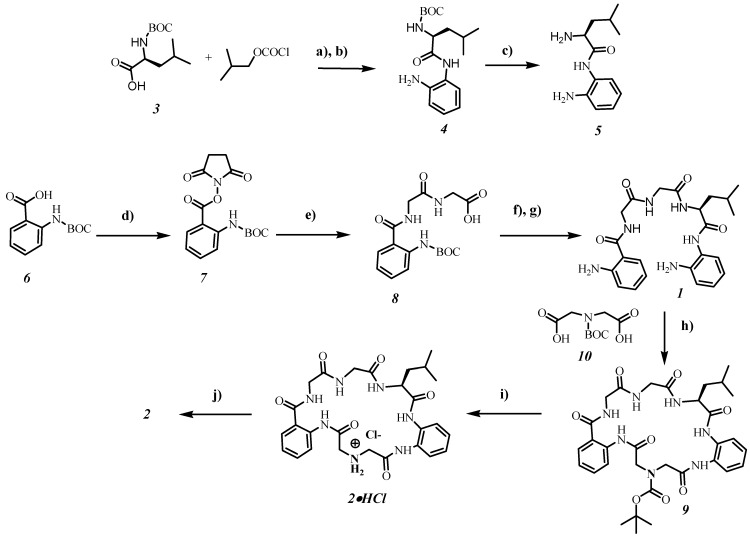
Synthesis of receptors **1** and **2**.

In Figure 2 the synthesis of receptors **1** and **2** is shown. Target compound **1** was constructed by coupling of protected amine **8** with diamine **5**. The deprotection step with trifluoroacetic acid yielded receptor **1** in 37% overall yield based on starting compound **6**. The macrocyclization step with the help of Boc-protected iminodiacetic acid **10** towards compound **9** was carried out at high dilution conditions giving the product in 36% yield. Deprotection of macrocycle **9** with trifluoroacetic acid gave only traces of receptor **2** and a mixture of different acyclic side-products, according to the NMR measurements. The best method for the deprotection step was the treatment of **9** with diethyl ether saturated with hydrogen chloride. By using this method, receptor **2** was obtained in 15% overall yield.

*Reagents and conditions*: (a) diisopropylethylamine (DIPEA); (b) 1,2-phenylenediamine; (c) CHCl_3_/Et_2_O saturated with HCl, then NaHCO_3_; (d) 1-ethyl-3-(3-dimethylaminopropyl)carbo-diimide (EDC), 4-(dimethylamino)-pyridine (DMAP), thriethylamine, *N*-hydroxysuccinimide (NHS); (e) DIPEA, diglycine; (f) EDC, DIPEA, 1-hydroxybenzotriazole (HOBt) and diamine **5**; (g) TFA in dichloromethane; (h) EDC, HOBt, DIPEA; (i) CHCl_3_/Et_2_O saturated with HCl; (j) NaHCO_3_ (5% aq.), extraction with ethyl acetate. BOC—*t*-butoxycarbonyl.

### 2.2. Anion Binding Studies

Binding properties of receptors towards different anions were investigated by UV-Vis, fluorescence and NMR titrations in an acetonitrile solution. The receptors bear an anthranilic acid moiety, which exhibits fluorescence emission at 400 nm upon excitation at 250 or 328 nm. Thus, we could use two complementary spectrophotometric methods to determine binding constants for anions. The fitting of binding isotherms was carried out with HypSpec Program [20]. As inferred from the dilution experiments the host molecules do not form associates at concentrations used in studies.

**Figure 3:**
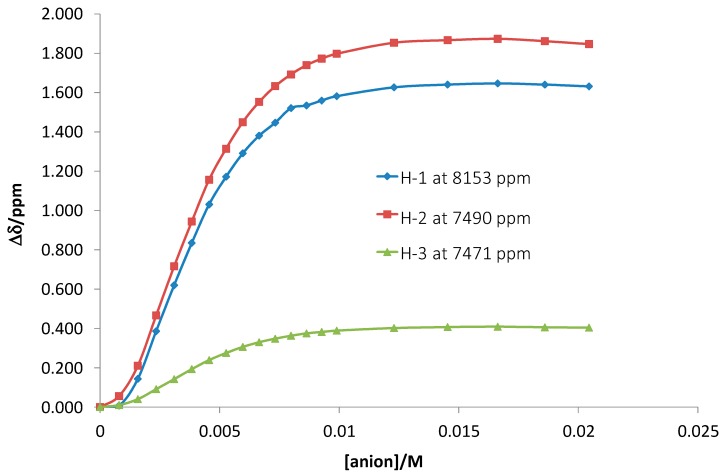
^1^H-NMR chemically induced resonance shifts upon addition of tetrabutylammonium dihydrogen phosphate to receptor **1**. H1 is the amide-NH of the anthranilic acid, H2 is the amide-NH connected to the phenylenediamine. H3 is the amide-NH of one of the amino acid residues.

Receptor **1** has an acyclic structure and it was expected from the beginning that it could bind all anions in question. However, chloride and nitrate induced very small changes in UV-Vis spectra so that fitting of the data was not possible. In spite of this fact, the chloride anion induced considerable changes in fluorescence titrations and the determined affinity constant was 520 M^−1^. Receptor **1** showed high affinity for dihydrogen phosphate, hydrogen sulfate and acetate. For the latter two anions we observed a stepwise binding mode—1:1 and 1:2 (receptor:anion). The Job’s plot analysis confirmed this stoichiometry (see Supporting information). Interestingly, receptor **1** binds two dihydrogen phosphate anions almost simultaneously, as inferred from the fitting model. The first binding event with a 1:1 stoichiometry is likely too weak to assess it by the curve-fitting analysis. Additional proof of a 1:2 binding mode was obtained from the NMR titration experiment. The affinity constant for the binding of two dihydrogen phosphate anions to receptor **1** was logβ_12_ = 8.98(10) (Figure 3). This value is in agreement with the value obtained by using spectrophotometric measurements. Interestingly, the strongest shifts were observed for amide protons H1 (the anthranilic acid fragment) and H2 (the phenylenediamine fragment) belonging to aromatic systems. Smaller shifts were detected for amide protons of the amino acids (e.g., H3).

**Figure 4:**
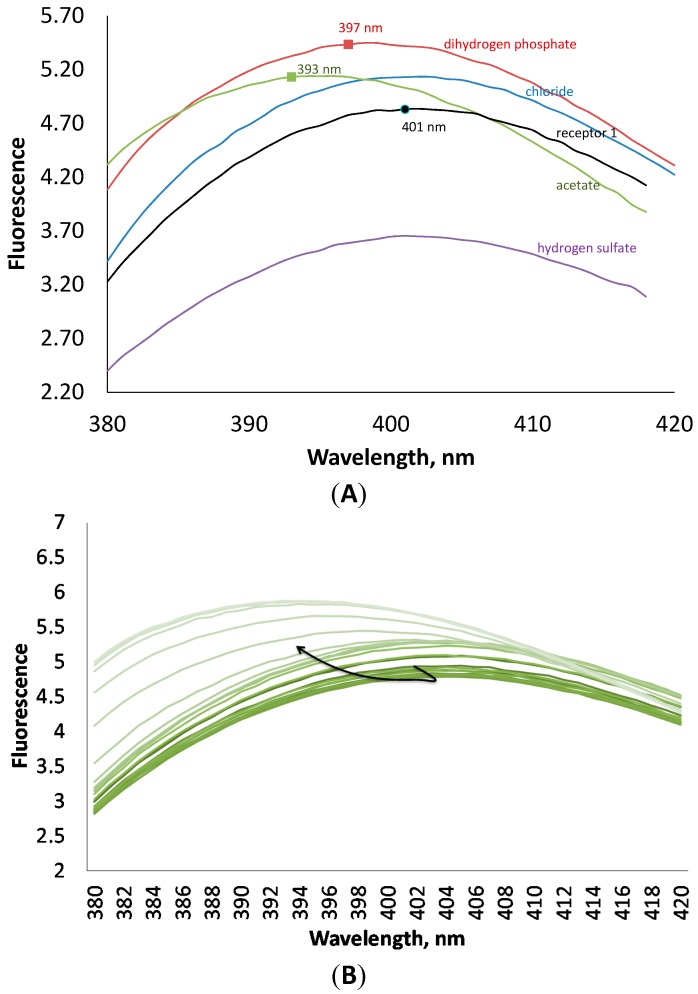
(**A**) Fluorescence spectra of receptor **1** at 10^−4^ M concentration in the presence of 4 equivalents of different anions in an acetonitrile solution. (**B**) Changes in fluorescence of **1** upon addition of tetrabutylammonium dihydrogen phosphate.

Binding constants obtained by fluorescence measurements were in a good agreement with those obtained by UV-Vis titrations (Table 1). As a rule, all anions enhance the fluorescence of **1**, except hydrogen sulfate. The latter anion quenches the fluorescence of the receptor. The addition of anions induced also a small hypsochromic shift (Figure 4). These interesting changes in fluorescence can be explained in terms of strong interactions through hydrogen bonds between the anions and the anthranilic acid moiety, which is responsible for fluorescence.

**Table 1:** Association constants log*K* of receptor **1** with different anions in an acetonitrile solution measured at 25 °C. *^a^* Small changes were observed upon the addition of the anion, so that it was not possible to fit the data; *^b^* Calculation of the first binding event was not possible due to the high fitting error.

Anion	UV-Vis	Fluorescence
Cl^−^	- *^a^*	*log*β*_11_* = 2.72 ± 0.01 *^a^*
CH_3_COO^−^	*logK_11_* = 5.51 ± 0.08 *logK_12_* = 3.99 ± 0.06	*logK_11_* = 5.00 ± 0.06 *logK_12_* = 3.93 ± 0.06
H_2_PO_4_^−^	*log*β*_12_* = 7.85 ± 0.01 - *^b^*	*log*β*_12_* = 7.95 ± 0.03 - *^b^*
HSO_4_^−^	*logK_11_* = 6.40 ± 0.08 *logK_12_* = 2.88 ± 0.06	*logK_11_* = 6.00 ± 0.04 *logK_12_* = 4.87 ± 0.05

According to the titration experiments, receptor **2** binds anions stepwise in 1:1 and 1:2 modes, except for chloride. This behavior is similar to that observed for receptor **1**. While for receptor **1** we observed almost no changes in UV-Vis titrations after addition of chloride, in case of the macrocyclic receptor chloride induced considerable changes in spectra so that we could determine the affinity constant. Interestingly the affinities of **1** and **2** for chloride are the same. All these facts indicate that the coordination of chloride induces considerable changes in the conformation of the macrocyclic receptor, likely orienting NH-donor groups towards the chloride anion. Because the affinity constant for chloride is much less, than that for phosphate, one can suggest that the orientation of NH-donors fits better the geometry of phosphate.

While the acyclic receptor did not show selectivity for a particular oxyanion, the macrocyclic receptor has a remarkable preference to bind dihydrogen phosphate selectively. This can be better seen from the comparison of the cumulative affinity constants for anions, e.g., for acetate logβ_12_ is 8.46, for dihydrogen phosphate logβ_12_ is 10.51 (Table 2). Thus, the macrocyclic structure allowed us to achieve selectivity for dihydrogen phosphate in an acetonitrile solution. Interestingly, hydrogen sulfate induced no changes in fluorescence but small changes in UV-Vis. By using the latter method, we were able to determine the affinity constant for hydrogen sulfate, which is two orders of magnitude lower than that for dihydrogen phosphate.

**Table 2:** Association constants log*K* of receptor **2** with different anions in an acetonitrile solution measured at 25 °C. *^a^* Small changes were observed upon the addition of the anion, so that it was not possible to fit the data.

Anion	UV-Vis	Fluorescence
Cl^−^	*logK_11_* = 2.70 ± 0.003	*logK_11_* = 2.72 ± 0.01
CH_3_COO^−^	*logK_11_* = 4.63 ± 0.006 *logK_12_* = 3.83 ± 0.09	*logK_11_* = 4.60 ± 0.10 *logK_12_* = 4.18 ± 0.03
H_2_PO_4_^−^	*logK_11_* = 6.42 ± 0.07 *logK_12_* = 4.09 ± 0.07	*logK_11_* = 6.00 ± 0.05 *logK_12_* =4.16 ± 0.05
HSO_4_^−^	*logK_11_* = 3.94 ± 0.02 *logK_12_* = 2.84 ± 0.05	- *^a^*

**Figure 5:**
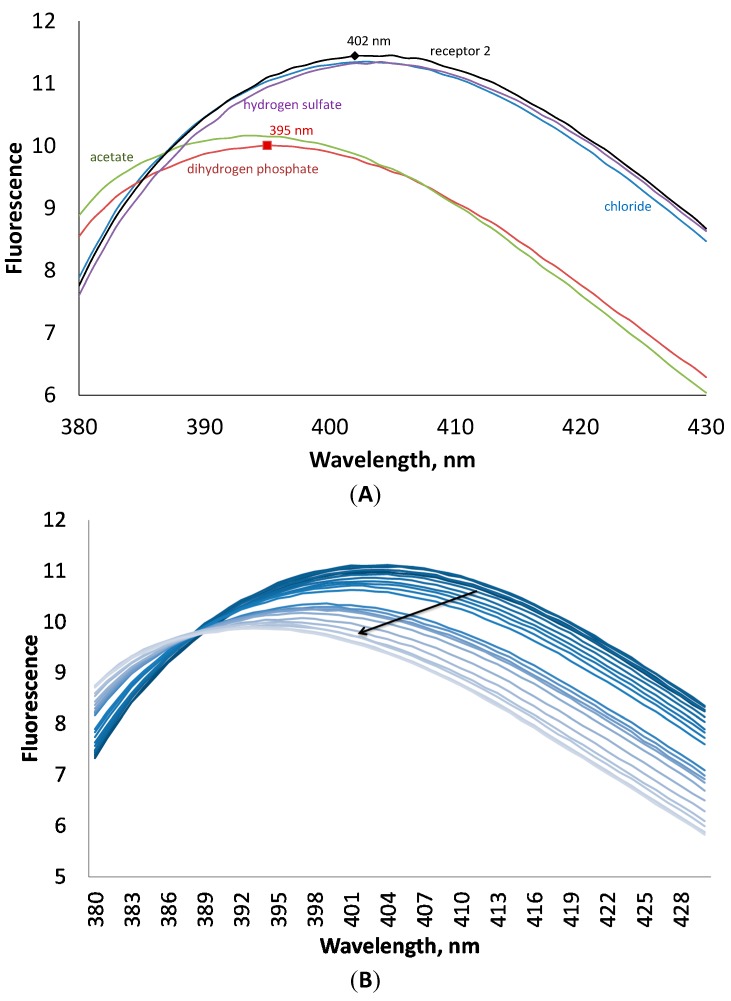
(**A**) Fluorescence spectra of receptor **2** at 10^−4^ M concentration in the presence of 4 equivalents of different anions in an acetonitrile solution. (**B**) Changes in fluorescence of **2** upon addition of tetrabutylammonium dihydrogen phosphate.

According to fluorescence measurements, receptor **2** demonstrated a different fluorometric answer towards anions, as compared to receptor **1**. The addition of anions induced quenching of fluorescence. As can be seen in Figure 5, the addition of four equivalents of chloride and hydrogen sulfate has almost no influence on the fluorescence of the host, while acetate and dihydrogen phosphate decrease the fluorescence intensity and shift the maximum towards lower wavelength numbers on 7 nm. This interesting behavior of the receptor to shift the fluorescence maximum upon addition of anions can be investigated in future studies in details for the design of ratiometric probes for anions [21].

**Figure 6:**
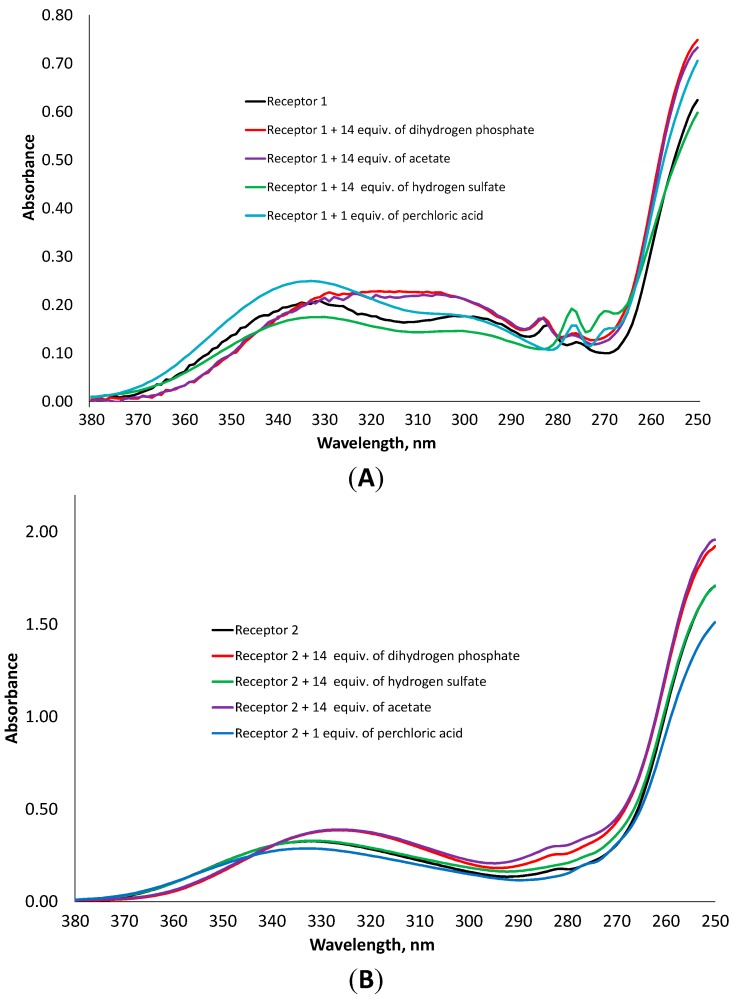
Comparison of UV-Vis spectra of receptor **1** (**A**) and **2** (**B**) in the presence of one equivalent of HClO_4_ and 14 equivalents of dihydrogen phosphate, hydrogen sulfate and acetate, respectively.

The property of receptors **1** and **2** to bind two anions, though the cavity does not allow this, is rather unusual. However, this behavior can have three different reasons. First, the coordination of two dihydrogen phosphate anions can be explained by dimerization of the phosphate anion in the presence of a synthetic receptor through the P-O…H-O-P hydrogen bond. Such a precedent has been already reported in the literature [22]; Second, HSO_4_^−^ and H_2_PO_4_^−^ anions have protons, which can be transferred to free amino groups during the anion coordination enabling the binding of the second anion to the positively charged receptor. This will lead to the formation of very stable complexes, in which the receptor and an anion have both electrostatic and hydrogen bonding interactions; Third, the receptor has a conformation, in which two pairs of donor NH-groups are oriented in different directions so that two anions could be bound from different sides. Such a situation has been observed for the urea-based receptors, which bind two acetate anions stepwise [23] and for the phosphate receptor reported by us earlier [24]. In order to understand the binding of anions by the synthesized receptors, we added to a solution of the receptor one equivalent of perchloric acid in acetonitrile, measured UV-Vis spectrum and compared this spectrum with those obtained after addition of different oxyanions. According to UV-Vis and fluorescence measurements, the receptors do not coordinate the perchlorate anion, therefore the addition of perchloric acid can be considered as a pure protonation effect. As can be seen in Figure 6, the spectrum of the protonated receptor **1** has the same structure as that obtained by the addition of hydrogen sulfate. An increase in 277 nm band and a decrease in 285 nm band were observed. In case of receptor **2**, the addition of the acid or hydrogen sulfate leads to the disappearance of a small shoulder at 282 nm, while the addition of dihydrogen phosphate or acetate induces a considerable hypsochromic shift.

These facts indicate that both receptors are protonated after addition of HSO_4_^−^, but not after addition of dihydrogen phosphate, acetate or chloride. The interaction of hydrogen sulfate with the receptors leads to the protonation so that the resulting SO_4_^2−^ anion can be also located outside the cavity. Such a situation was often observed in the solid state for positively-charged cryptand receptors [25]. Analysis of NMR spectra of receptor **1** and **2** in the presence of anions in a CD_3_CN solution supports this hypothesis. The binding of dihydrogen phosphate or acetate leads to sharpening of the proton signals, while binding of hydrogen sulfate induces broadening of both NH and CH signals (see Supporting Information).

**Figure 7:**
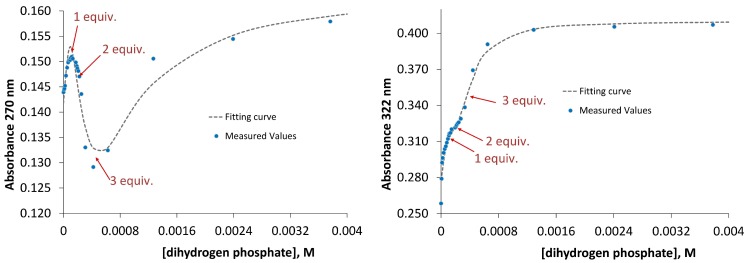
UV-Vis titration data and fitting curves obtained for the addition of dihydrogen phosphate to the mixture of receptor **1** (**left**) and receptor **2** (**right**) with one equivalent of perchloric acid.

In order to explain a 1:2 stoichiometry of oxyanion binding we proposed that both receptors possess a specific conformation that enables binding of two anions. To prove our suggestion we protonated the receptors with one equivalent of perchloric acid and titrated the resulting salts with dihydrogen phosphate anion. If a receptor is able to accommodate only one anion then the corresponding positively charged receptor should bind two dihydrogen phosphate anions because it is added in excess and one anion will be located in the binding cavity and the other—outside the cavity stabilized by electrostatic interactions. If a receptor is able to accommodate two anions then the corresponding positively charged receptors should bind three dihydrogen phosphate anions. Exactly the latter situation we observed in UV-Vis titrations of salts **1**•HClO_4_ and **2**•HClO_4_ with tetrabutylammonium dihydrogen phosphate. As can be seen in Figure 7 the titration curve changes its direction almost exactly at points 1, 2 and 3 equivalents of dihydrogen phosphate, respectively. Fitting of the experimental data with HypSpec Program resulted in cumulative association constants β_13_(for **1**) = 12.82 ± 0.01 and β_13_(for **2**) = 13.69 ± 0.01 (Figure 7).

**Figure 8:**
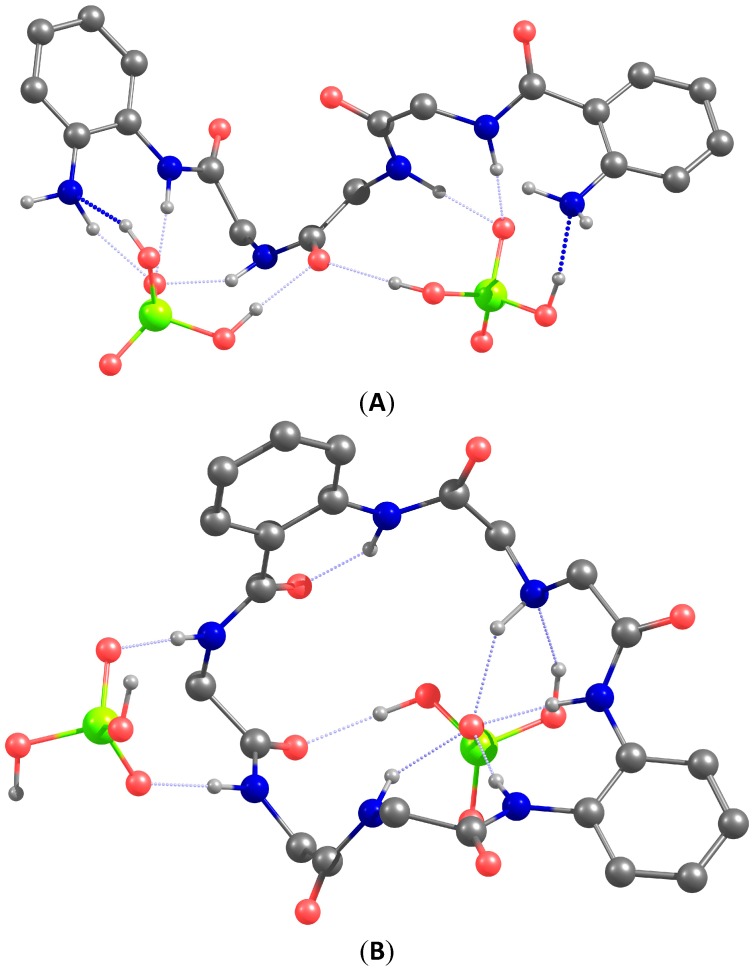
Calculated structures of complexes **1•**(H_2_PO_4_^−^)_2_ (**A**) and **2•**(H_2_PO_4_^−^)_2_ (**B**). The isopropyl substituent and most of the hydrogen atoms were omitted for clarity. The atoms are shown in different color Red: oxygen; green: phosphorus; blue: nitrogen; gray: carbon.

To get an understanding how two dihydrogen phosphate anions can be coordinated by our receptors we carried our DFT modeling of the complexes. The optimized structures of complexes **1•**(H_2_PO_4_^−^)_2_ and **2•**(H_2_PO_4_^−^)_2_ are depicted in Figure 8. The following conclusions can be drawn from the analysis of the structures of the optimized host-guest complexes. In the acyclic receptor one amino acid subunit bearing two NH-sites coordinates one oxygen atom of phosphate. This binding mode is similar to that of phosphate coordination by the kinase (Figure 1). Interestingly, in both complexes the amide oxygen atoms play a role of hydrogen bond acceptor, as well as amino groups, which facilitates the coordination of the protonated phosphate anion. Macrocyclic receptor has a rigid structure and coordinates two dihydrogen phosphate anions from different sides. One of the anions is stabilized only with two NH-sites. This coordination mode hints how two acetate or two sulfate anions can be bound to the receptor. Unexpected for us was the fact that the phenylenediamine subunit forms an almost planar arrangement of NH-sites, which bind one oxygen atom of the phosphate anions. This arrangement explains the affinity of the receptor for the chloride anion.

## 3. Experimental Section

### 3.1. General Information

All solvents were of reagent grade quality and purchased commercially. All starting materials were purchased from Sigma-Aldrich (Steinheim, Germany) and Fluka Chemical Co. (Steinheim, Germany) NMR spectra used in the characterization of products were recorded on an Avance 300 instrument (Bruker, Chemnitz, Germany). The NMR spectra were referenced to trace solvent peak, the spectroscopic solvents were purchased from Deutero GmbH (Kastellaun Germany). Mass spectra were recorded with Finnigan MAT SSQ 710 A (CI) and Finnigan MAT 95 (HRMS) (Regenesburg, Germany). Column chromatography was performed on Whatman silica gel 60 Å (230–400 mesh). UV/Vis spectra were recorder on Cary BIO 50 UV/Vis/NIR spectrometer (Varian, Chemnitz, Germany). N-BOC protected antranilic acid, *N*-hydroxysuccinimide derivative [26] and BOC-protected iminodiacetic acid [27] were prepared according to the literature procedures.

### 3.2. DFT Calculations

Molecular modeling calculations were performed using the DFT program “PRIRODA” [28]. A PBE functional which includes electron density gradient was used. TZ2p-atomic basis sets of grouped Gaussian functions were used to solve the Kohn—Sham equations. The criterion for convergence was a difference below 0.01 kcal/mol/Angstrom in the energy between two sequential structures. Various stationary points on potential energy surface (PES) were determined from analytical calculations of second energy derivatives (Hessian matrixes).

### 3.3. (R)-2-Amino-N-(2-aminophenyl)-4-methylpentanamide *(**5**)*

N-Boc-(l)-Leucine (200 mg, 0.86 mmol) and DIPEA (163 µL, 1 mmol) were dissolved in 30 mL of dry THF and the solution was cooled to −20 °C using an acetone-dry ice bath. Isobutyl chloroformate (136.2 µL, 1 mmol) was added at one portion and the solution was stirred for 40 min at this temperature. 1,2-Diaminobenzene (186 mg, 1.72 mmol) was dissolved in THF (20 mL) and added to the first solution at one portion. At the same time, the cooling bath was removed allowing the mixture to warm up during next 24 h. The resulting solution was evaporated and separated on a silicagel using dichloromethane-methanol 100:2 *v*/*v* mixture as eluent, R_f_ = 0.6 (second fraction). The purity of the product was controlled by: ^1^H-NMR (CDCl_3_) δ 8.04 (s, 1H), 7.17 (m, 1H), 7.07–6.95 (m, 1H), 6.79–6.63 (m, 2H), 5.17 (d, *J* = 7.4 Hz, 1H), 4.17 (dd, *J* = 49.2, 6.2 Hz, 1H), 3.97–3.75 (bs, 2H), 1.74 (m, 2H), 1.66–1.50 (m, 1H), 1.42 (s, Hz, 9H), 1.03–0.87 (m, 6H). ^13^C-NMR (CDCl_3_) δ 171.5, 156.3, 140.7, 127.1, 125.5, 123.6, 118.9, 117.3, 28.34, 24.85, 22.95, 22.06, 19.08, 18.89. Deprotection was accomplished by stirring the compound in ether (HCl gas saturated) solution (4 mL) at 0 °C for 24 h. The white precipitate was washed with ether and dried yielding **5**·(HCl)_2_. The salt was dissolved in distilled water and basic ion exchange resin was added until the pH of the solution became 8–9. The resin was filtered off, and the solution was lyophilized giving **5** as a free amine (96 mg ,50%). ^1^H-NMR (CDCl_3_) δ 7.27 (d, *J* = 6.3 Hz, 1H), 7.10–6.93 (m, 1H), 6.85–6.70 (m, 2H), 6.62–6.27 (m, 1H), 3.94 (d, *J* = 6.7 Hz, 2H), 3.65 (bs, 2H), 2.16 (s), 2.06–1.84 (m, 2H), 0.95 (d, *J* = 6.7 Hz, 6H). ^13^C-NMR (CDCl_3_) δ 265.8, 154.9, 140.1, 126.4, 124.7, 119.3, 117.6, 71.6, 30.9, 27.8, 18.6. ESI-MS(+): *m*/*z* [M+H]^+^ 222.1. Anal. Calcd: C, 65.13%; H, 8.65%; N, 18.99%. Found: C, 65.40%; H, 8.66%; N, 19.05%.

### 3.4. ((2-[(tert-Butoxy)carbonyl]amino)phenyl)formamido-Gly-Gly-OH *(**8**)*

N-Boc antranilic acid (2.00 g, 8.42 mmol), EDC hydrochloride (1.80 g, 9.43 mmol), *N*-hydroxysuccinimide (1.09 g, 9.43 mmol), DMAP (30 mg, 3 mol %) and triethylamine (1.32 mL, 9.43 mmol) were mixed in DCM (100 mL) and stirred for 2 h, followed by evaporation of solvent at 40 °C on a rotary evaporator. The solid was purified on a short silica-gel column using dichloromethane-methanol 100:5 *v*/*v* mixture as an eluent (R_f_ = 0.9), yielding 1.67 g (5 mmol, 60%) of *N*-hydroxy-succinimide derivative. The obtained product was dissolved in DMF (30 mL) and added to a round bottom flask containing diglycine (0.66 g, 5 mmol) and DIPEA (1 mL, 6 mmol) dissolved in a mixture of methanol (12 mL), ethyl acetate (6 mL) and water (6 mL). The mixture was stirred for 48 h, evaporated at 60–70 °C. The oily residue was dissolved in DCM (100 mL) and water (100 mL) was added. After 30 min stirring of the solution the white crystals of product precipitated, which were subsequently filtered off yielding 730 mg (41%) of **8**. If the product do not precipitate the mixture can be purified using column chromatography by subsequent washing of the column with 100:5 and then 80:20 *v*/*v* dichloromethane-methanol mixtures.^1^H-NMR (DMSO-*d*_6_) δ ppm 7.08 (t, *J* = 7.30 Hz), 7.48 (t, *J* = 7.64 Hz), 7.80 (d, *J* = 7.59 Hz), 8.58–8.05 (m), 9.03 (s), 10.70 (s), 3.84 (dd, *J* = 35.67, 5.21 Hz), 3.68–2.76 (m), 1.46 (s). ^13^C-NMR (300 MHz, MSO-*d*_6_) δ ppm 171.05; 168.82; 168.53; 152.00; 139.69; 132.21; 128.24; 121.20; 118.70; 118.30, 79.67; 27.84. ESI-MS(+): *m*/*z* [M+H]^+^ 352.2. Anal. Calcd: C, 54.69%; H, 6.02%; N, 11.96%. Found: C, 54.15%; H, 6.12%; N, 12.05%.

#### 3.4.1. ((2-[(*tert*-Butoxy)carbonyl]amino)phenyl)formamido-Gly-Gly-Leu-(2-aminophenyl)amine (**1**)

Compound **8** (750 mg, 2.14 mmol) was dissolved in dry dichloromethane (150 mL) containing EDC hydrochloride (490 mg, 2.56 mmol), HOBt (346 mg, 2.56 mmol), and DIPEA (845 µL, 5.12 mmol), and the solution was stirred for 1 h at 0 °C. Compound **5** (566 mg, 2.56 mmol) was added to the solution and the stirring was continued for 24 h at room temp. The solvent was evaporated and the residue purified using column chromatography in 100:10 *v*/*v* dichloromethane-methanol mixture yielding crude BOC-protected amine. Deprotection was performed by stirring the product in trifluoroacetic acid‒dichloromethane mixture (1:3 *v*/*v*) for 3 h, followed neutralization with Na_2_CO_3_, which resulted in precipitation of white powder. The suspension was extracted by ethyl acetate and purified using column chromatography (eluent: ethyl acetate-methanol 100:5 *v*/*v*) yielding 900 mg (92%) of **1**. ^1^H-NMR (DMSO-*d*_6_) δ 9.20 (s), 8.54 (t, *J* = 5.7 Hz), 8.21 (dd, *J* = 14.0, 8.4 Hz), 8.01 (d, *J* = 7.6 Hz), 7.55 (d, *J* = 7.9 Hz), 7.15 (t, *J* = 7.7 Hz), 7.10–7.01 (m), 6.92 (t, *J* = 7.6 Hz), 6.77–6.62 (m), 6.52 (ddd, *J* = 12.6, 6.9, 2.9 Hz), 4.79 (s), 4.44 (q, *J* = 7.4 Hz), 4.03 (q, *J* = 7.1 Hz), 3.92–3.65 (m), 2.09 (s), 1.99 (s), 1.62 (dt, *J* = 24.2, 6.7 Hz), 1.17 (t, *J* = 7.1 Hz), 0.89 (dd, *J* = 9.1, 6.3 Hz). ^13^C-NMR (DMSO-*d*_6_) δ 174.0, 173.4, 172.7, 172.2, 150.4, 141.7, 133.7, 129.4, 128.7, 127.9, 125.2, 120.7, 120.1, 119.0, 118.4, 117.5, 116.3, 116.2, 61.6, 53.9, 44.5, 43.8, 41.6, 25.9, 23.5, 21.8, 18.4, 14.5. ESI-MS(+): *m*/*z* [M+H]^+^ 455.0. Anal. Calcd: C, 60.78%; H, 6.65%; N, 18.49%. Found: C, 60.60%; H, 6.66%; N, 18.47%.

#### 3.4.2. (17*S*)-17-(2-Methylpropyl)-2,5,8,15,18,21,24-heptaazatricyclo[24.4.0.09,14]triaconta-1(30), 9(14),10,12,26,28-hexaene-3,7,16,19,22,25-hexone (**2**)

Boc-Protected iminodiacetic **10** (62 mg, 0.264 mmol) was dissolved in dry DCM (10 mL), cooled to 0 °C, and to the solution were added HOBt (72 mg, 0.53 mmol), DIPEA (86 µL, 0.53 mmol) and EDC (101 mg, 0.53 mmol). The mixture was stirred at this temperature for 1 h and poured to the solution of **1** (100 mg, 0.22 mmol) in dry DCM (150 mL). The reaction was stirred additional 48 h at room temp. and the solvent was evaporated. First a bluish fluorescent spot (under UV lamp) was separated on a silicagel column using ethyl acetate-methanol 100:5 *v*/*v* mixture as eluent yielding compound **9** in 36% yield. This compound was further deprotected by stirring it at 0 °C for 2 h and then overnight at room temperature in the mixture of chloroform-ether (HCl saturated), 5 and 10 mL, respectively. The precipitate formed was filtered off and washed with ether yielding salt **2•**HCl. The conversion to the free base was accomplished by dissolving the salt in a NaHCO_3_ (sat.)‒ethyl acetate mixture followed by subsequent extraction with ethyl acetate five times and evaporation. Compound **2** was obtained after silica gel column chromatography using ethyl acetate-methanol 85:15 *v*/*v* mixture as eluent with 15% yield. ^1^H-NMR (CD_3_OD) δ 7.65–7.61 (m, 1H), 7.51 (dd, *J* = 8.0, 1.3 Hz, 1H), 7.41 (dd, *J* = 7.8, 1.5 Hz, 1H), 7.21–7.13 (m, 3H), 6.75–6.70 (m, 1H), 6.59 (t, *J* = 7.5 Hz, 1H), 6.08 (t, *J* = 1.9 Hz, 1H), 4.58 (m, 1H), 4.07–3.91 (m, 2H) 3.71 (s, 2H), 3.50 (s, 2H) 3.47 (s, 2H), 1.78–1.74 (m, 3H), 0.94 (s, 3H), 0,93 (s, 3H). ESI-MS(+): *m*/*z* [M+H]^+^ 552.1. Anal. Calcd: C, 58.79%; H, 6.03%; N, 17.78%. Found: C, 58.93%; H, 5.98%; N, 17.69%.

## 4. Conclusions 

In summary, we have designed and synthesized two model receptors with a GGL binding unit that is found in active sites of kinases and this sequence is responsible for recognition of phosphate residues. Additional feature of our design has been the presence of free amino groups that serve as hydrogen-bond-acceptor groups and facilitate coordination of protonated anions, such as dihydrogen phosphate. Anion binding properties of two new receptors have been investigated with the help of UV-Vis, fluorescence and NMR titration methods in an acetonitrile solution. These complementary methods have produced in most cases identical results. The acyclic receptor has demonstrated a general selectivity for oxyanions over chloride and nitrate with high affinity constants. Stepwise 1:1 and 1:2 binding modes have been observed for acetate, hydrogen sulfate and dihydrogen phosphate. A remarkable preference to bind dihydrogen phosphate over acetate and hydrogen sulfate has been observed for the macrocyclic receptor. Comparison of the UV-Vis spectra of the host-guest complexes with the spectra of receptors protonated by perchloric acid has provided an evidence that protonation of free amino groups induces only hydrogen sulfate, while other anions are bound through hydrogen bonds. Fluorometric measurements have revealed a difference in responses of the acyclic and macrocyclic receptors towards anions. Addition of strongly coordinating anions to the solution of the acylic receptor has induced an increase in intensity, while in the case of the macrocyclic receptor a decrease of intensity has been detected. For both receptors a 5–8 nm shift of fluorescence maximum has been observed upon interaction with acetate and dihydrogen phosphate. DFT quantum chemical calculations have been carried out to understand the interaction of the receptors with dihydrogen phosphate in the complexes. Studies of anions binding properties of the reported receptors in an aqueous solution at pH values, at which the receptors are protonated, are in progress.

## Supplementary Materials

Supplementary materials can be accessed at: http://www.mdpi.com/1420-3049/20/02/3354/s1.
