# Supplementary material for: Investigation of Structural Mimetics of Natural Phosphate Ion Binding Motifs
Source: Molecules. 2015 Feb 16;20(2):3354–70. doi: 10.3390/molecules20023354 (PMC6272573; doi:10.3390/molecules20023354)
Supplement: Supplementary file 1 [file molecules-20-03354-s001.pdf]

Supplementary Materials

NMR spectra of the synthesized compounds

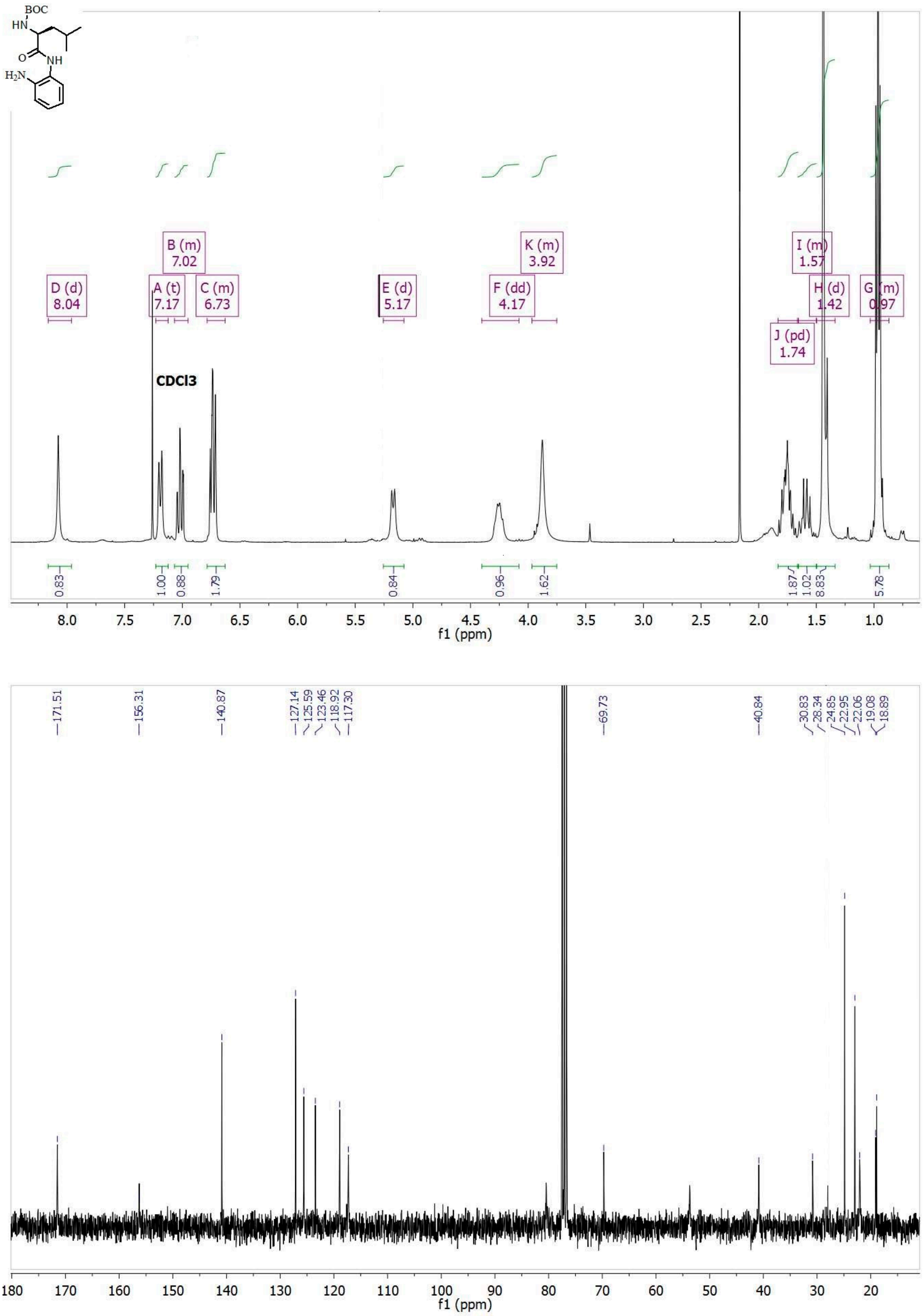

Figure S1. ^{1}H- and ^{13}C-NMR spectra of compound **4** in $CDCl_3$.

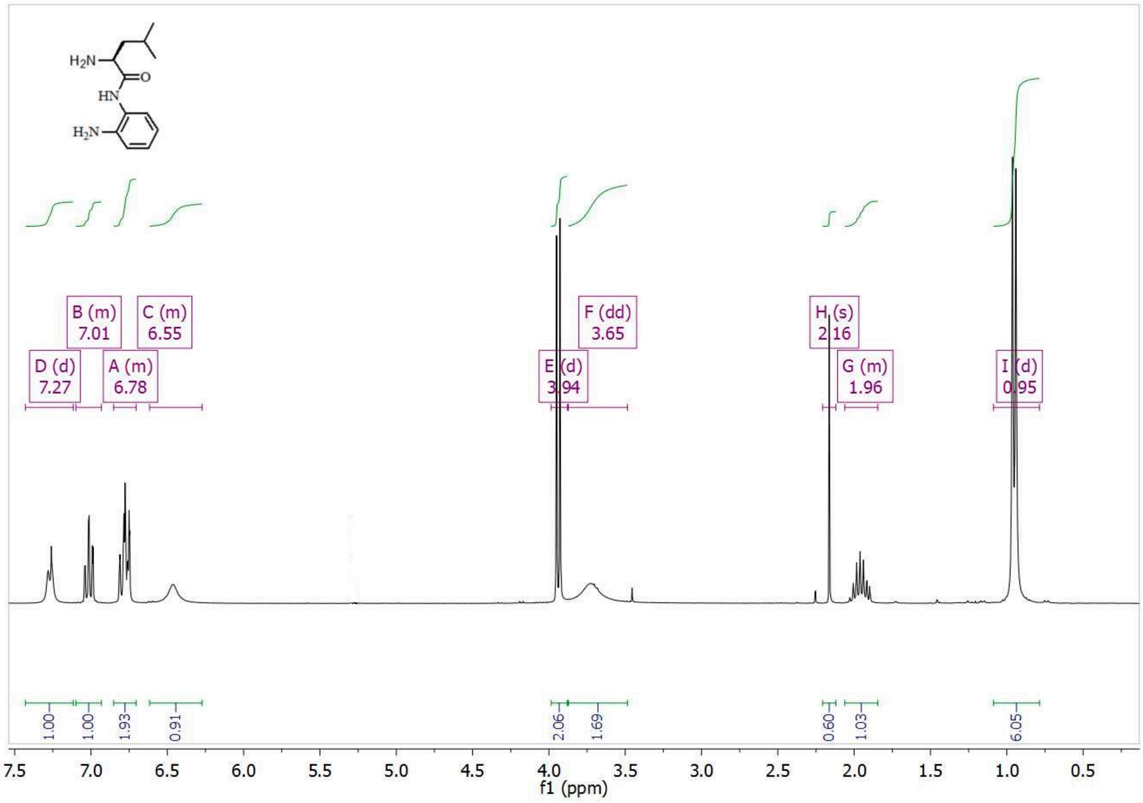

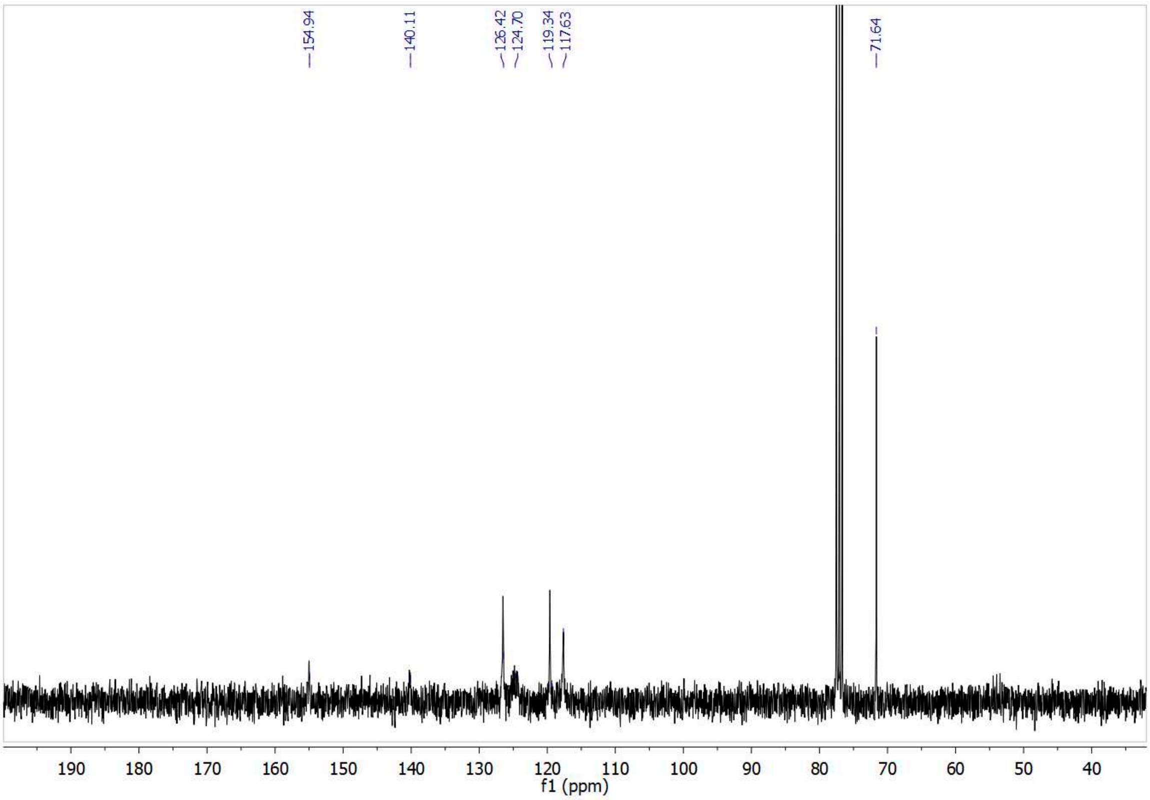

Figure S2. ^{1}H- and ^{13}C-NMR spectra of compound **5** in $CDCl_3$.

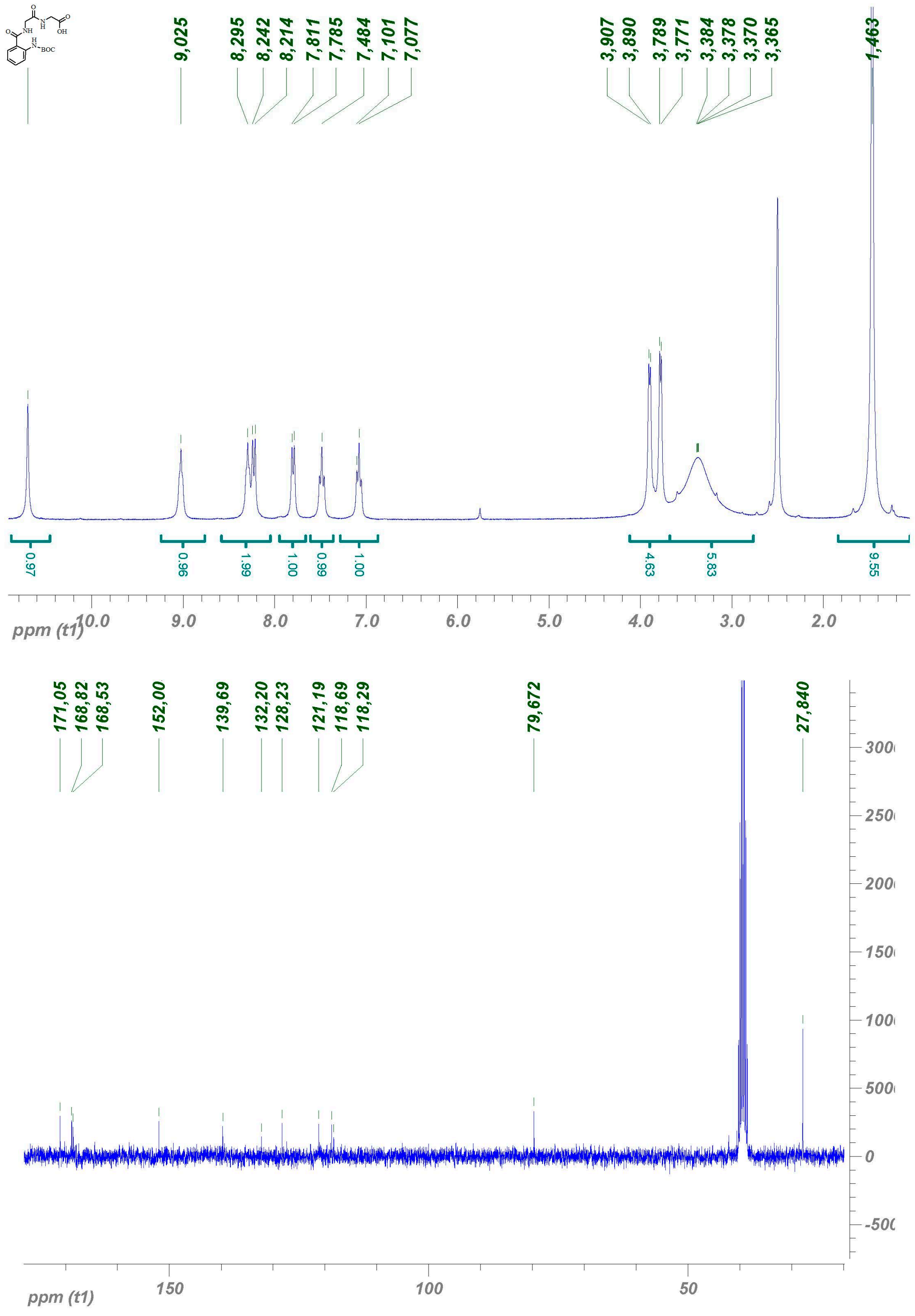

Figure S3. ^{1}H- and ^{13}C-NMR spectra of compound **6** in DMSO-d_6.

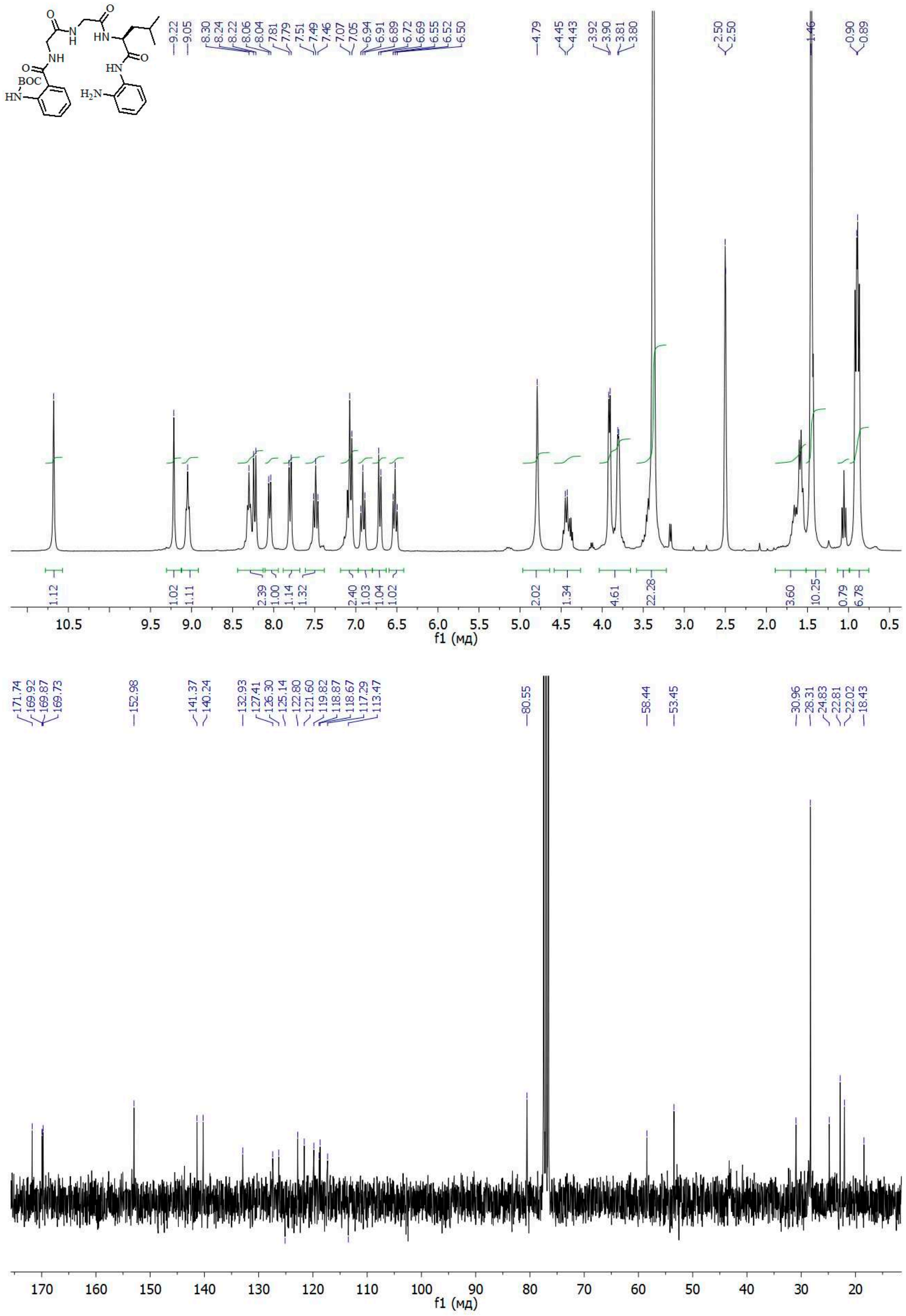

Figure S4. ^{1}H- and ^{13}C-NMR spectra of the BOC-protected receptor **1** in DMSO-d_6.

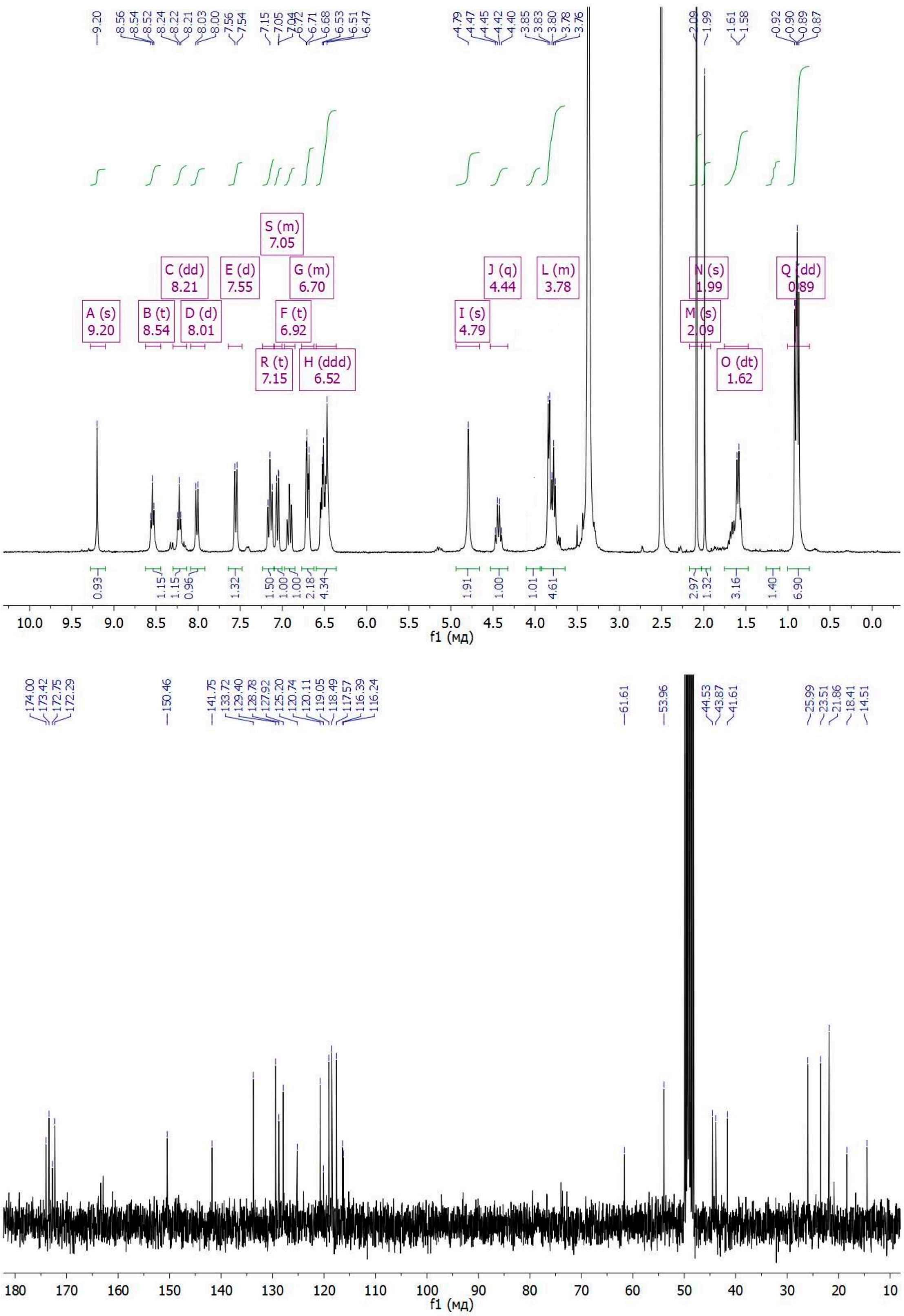

Figure S5. ^{1}H- and ^{13}C-NMR spectra of **1** in DMSO-d_6.

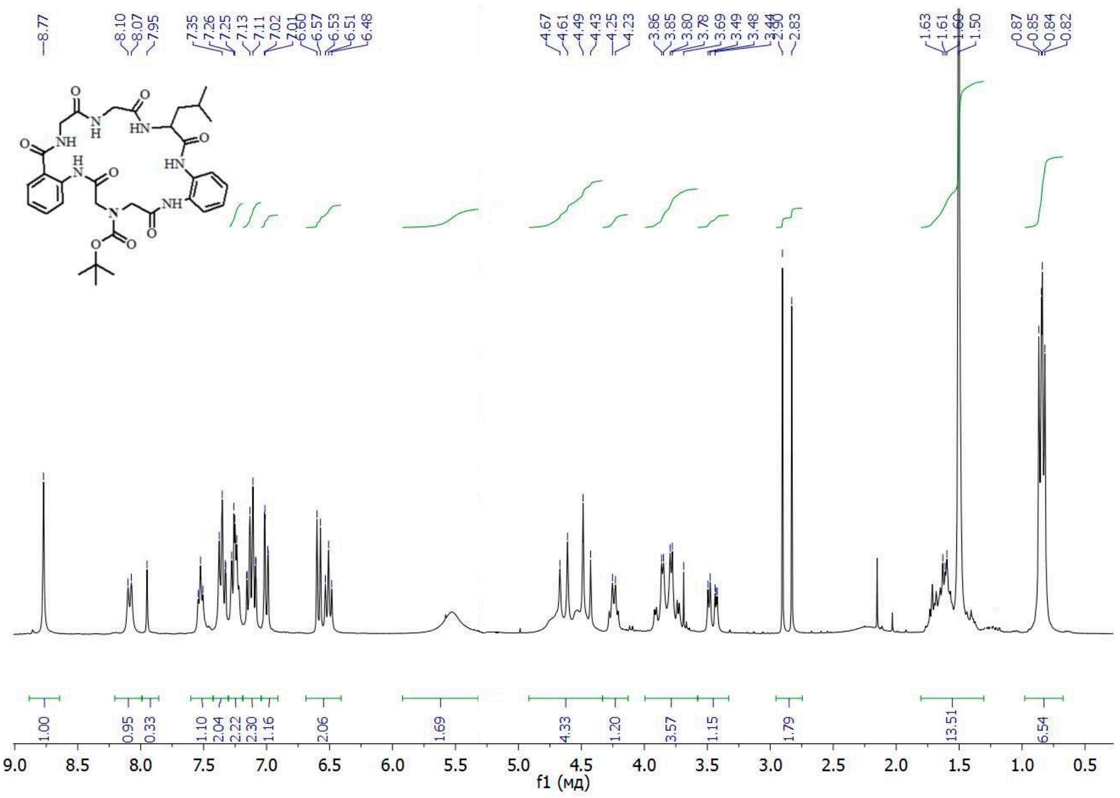

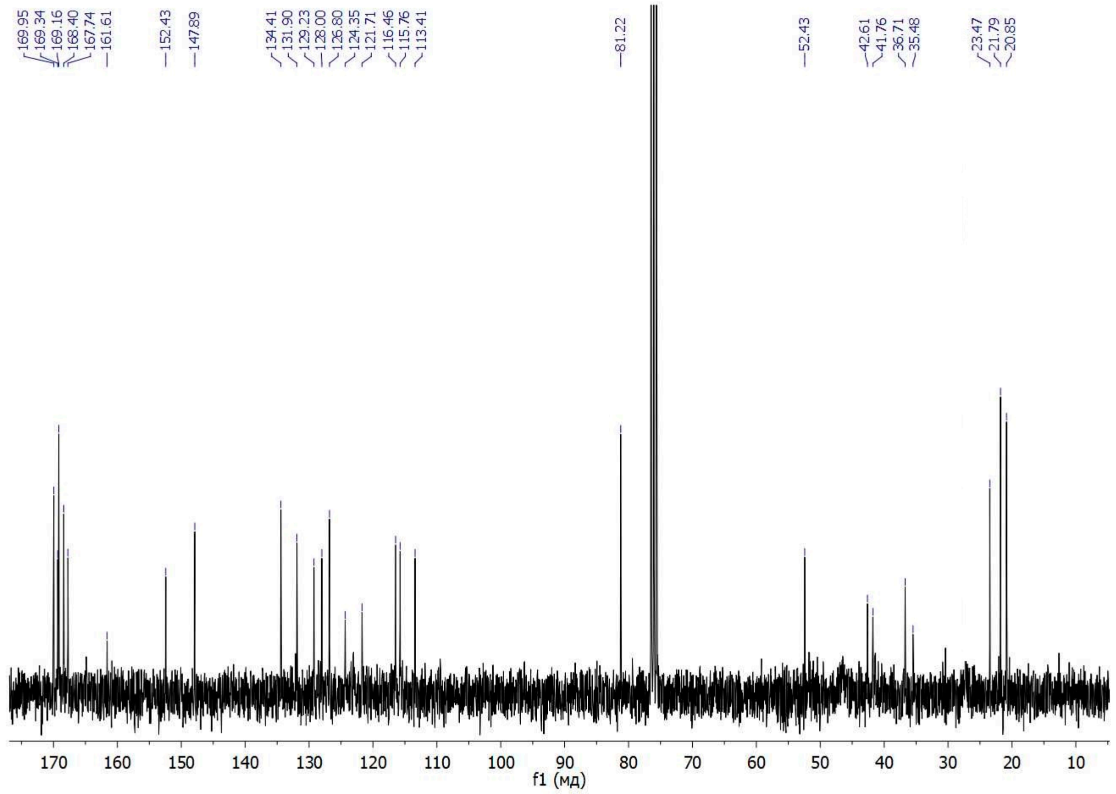

Figure S6. ^{1}H- and ^{13}C-NMR spectra of compound **9** in $CDCl_3$.

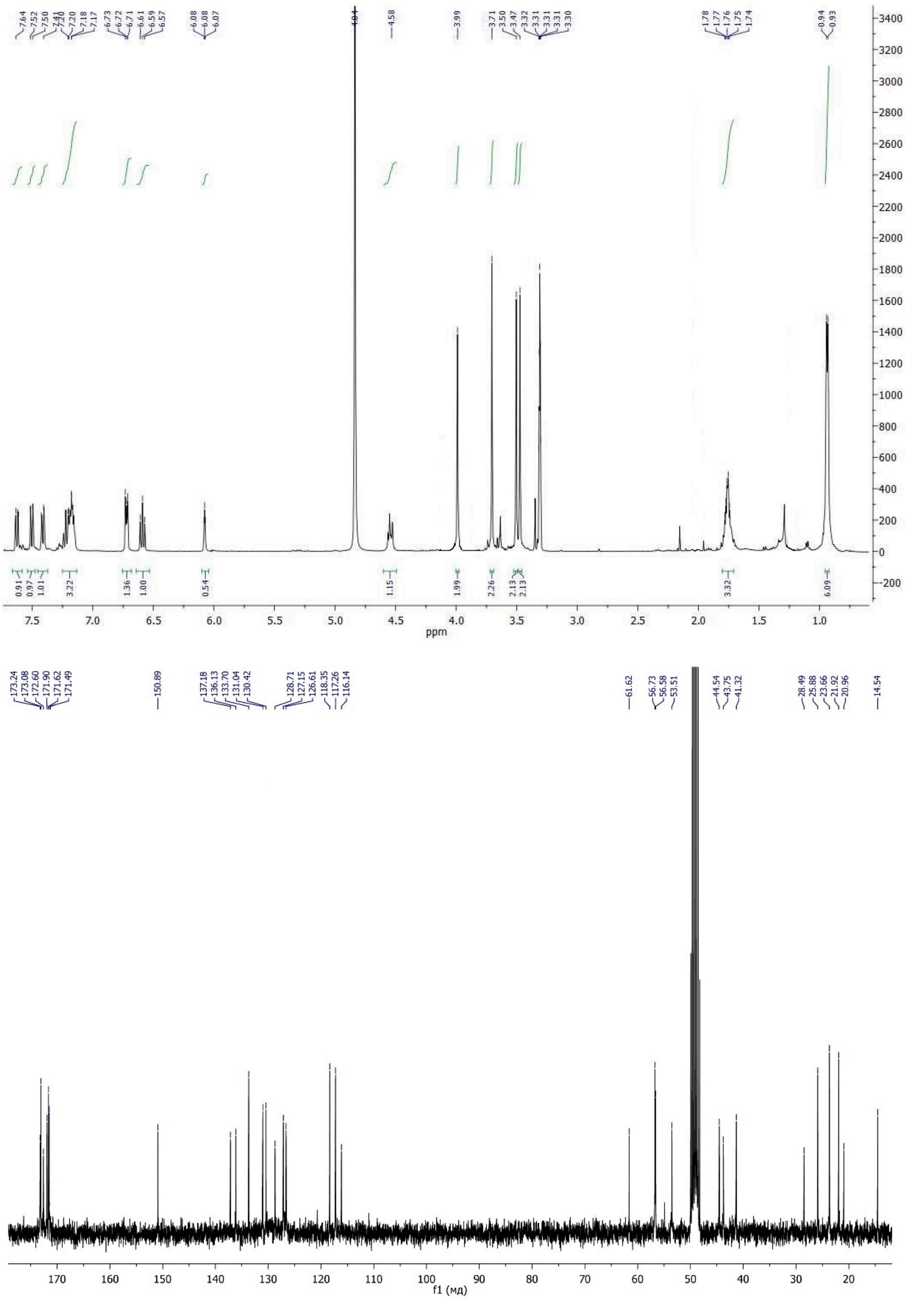

Figure S7. ^{1}H- and ^{13}C-NMR spectra of receptor **2** in CD_3OD.

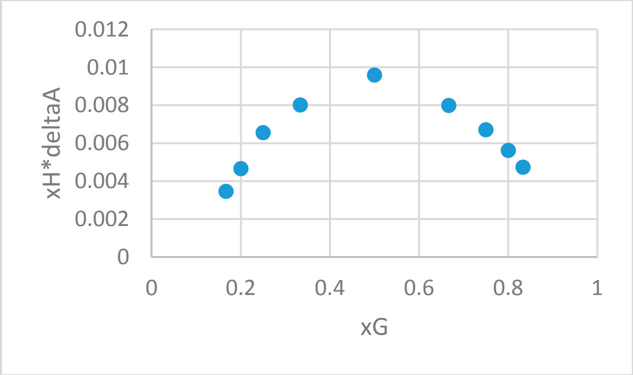

Figure S8. Typical Job's plot for binding of chloride, showing a 1:1 stoichiometry.

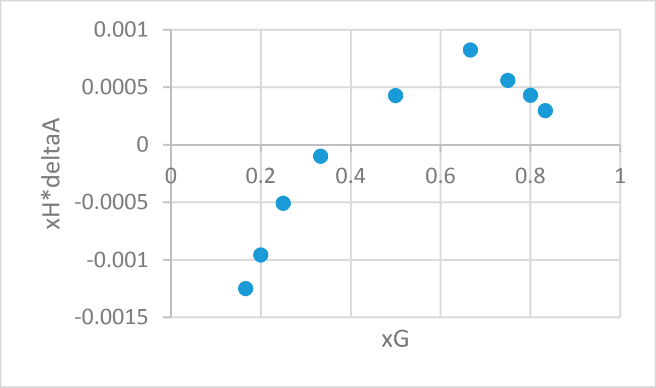

Figure S9. Typical Job's plot for binding of acetate, dihydrogen phosphate and hydrogen sulfate, showing a 1:2 stoichiometry.

UV-Vis and Fluorescence Titrations

Anion titrations were carried by a stepwise addition of 10^{-2}–10^{-3} M stock solution of guest to a 10^{-4} M stock solution of a receptor. The experimental data were fitted to binding modelsby using the whole spectrum with the help of the HypSpec program. All the titration experiments were carried out in HPLC grade acetonitrile solution.

Table S1. Changes UV-Vis spectra of receptor **1** upon addition of anions (**left**) and the data fitting (**right**).

$H_2PO_4^-$	
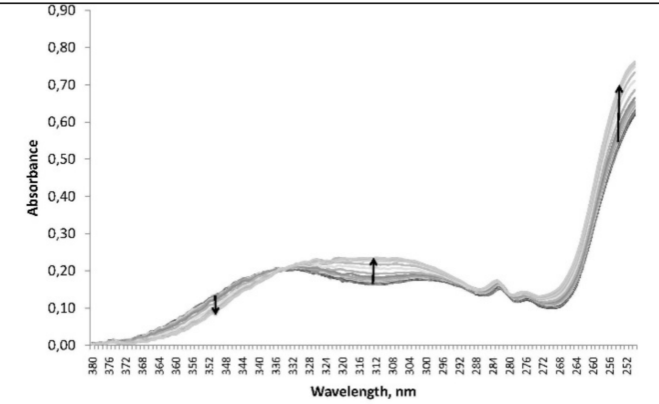	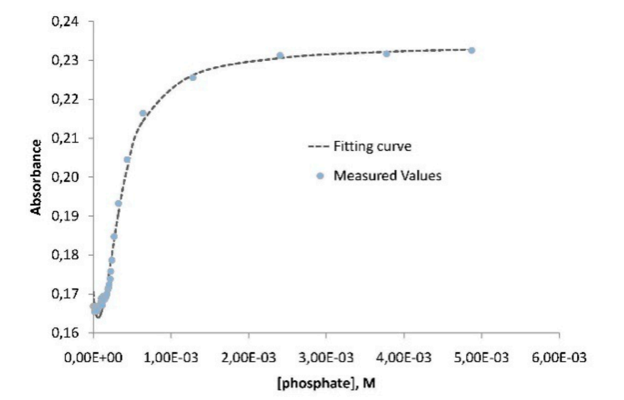

Table S1. *Cont.*

HSO$_4^-$	
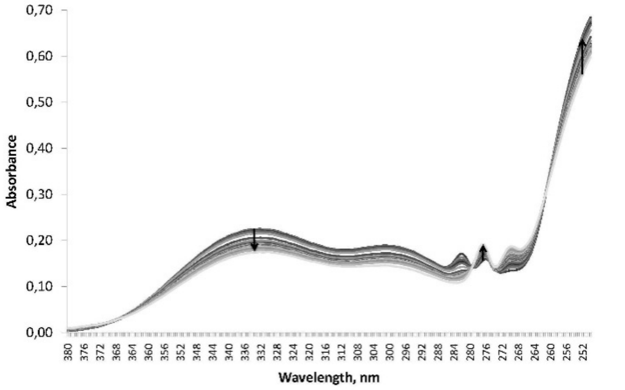	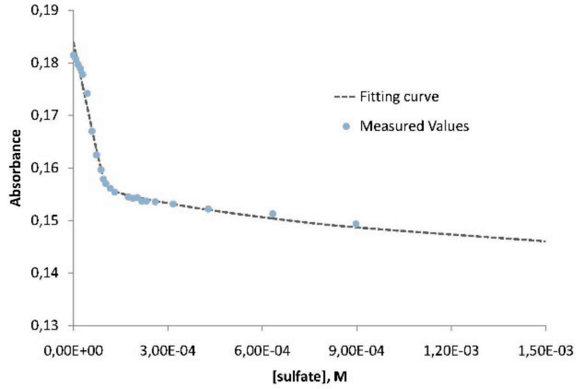
**CH$_3$COO$^-$**	
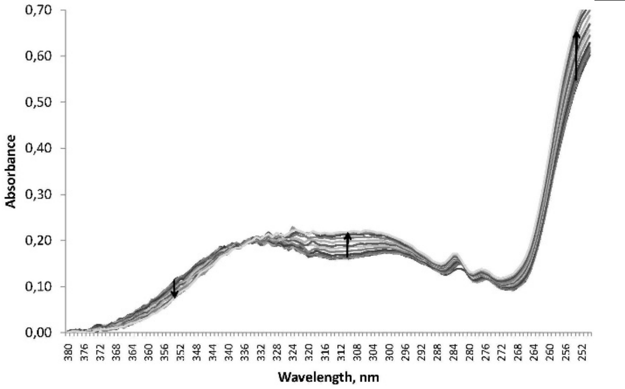	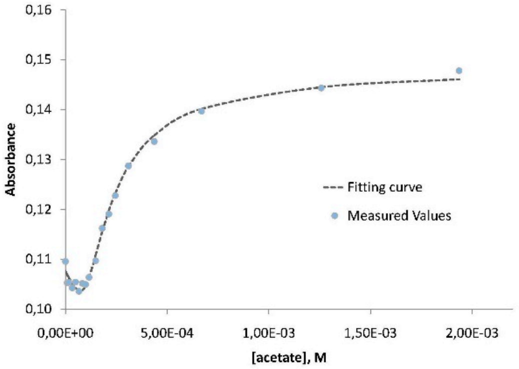

Table S2. Changes in fluorescence spectra of receptor **1** upon addition of anions (**left**) and the data fitting (**right**).

H$_2$PO$_4^-$	
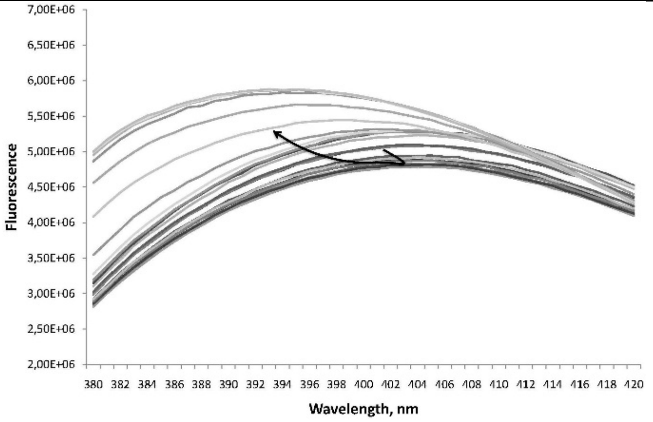	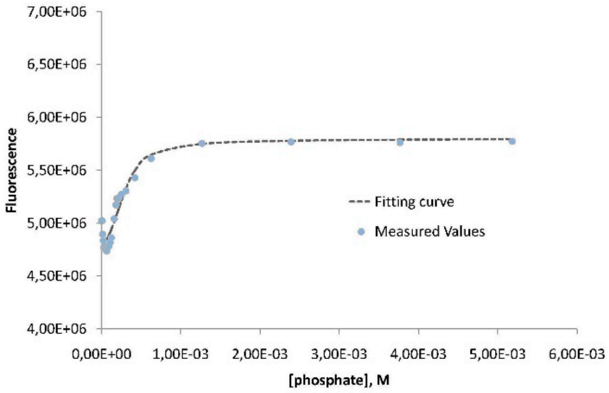

Table S2. *Cont.*

HSO_4^-

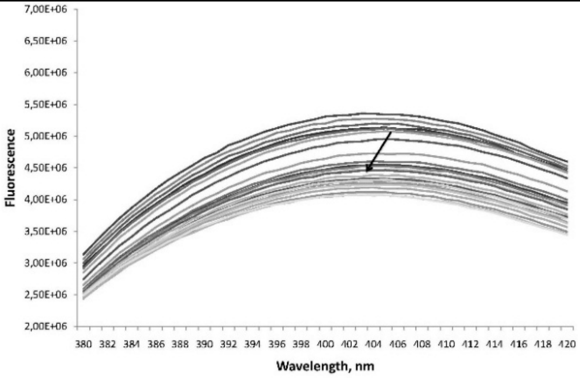

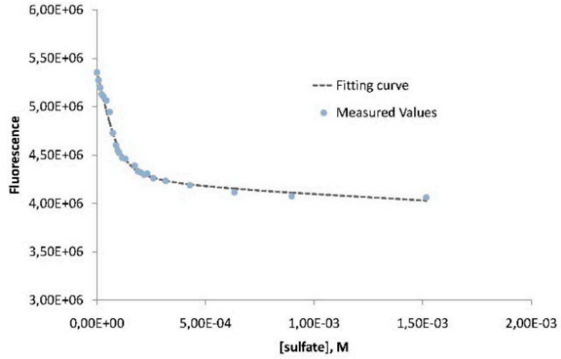

CH_3COO^-

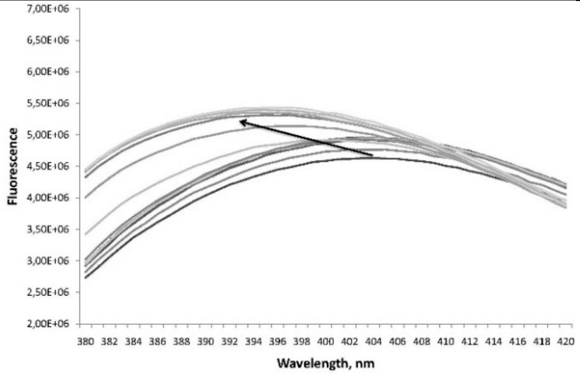

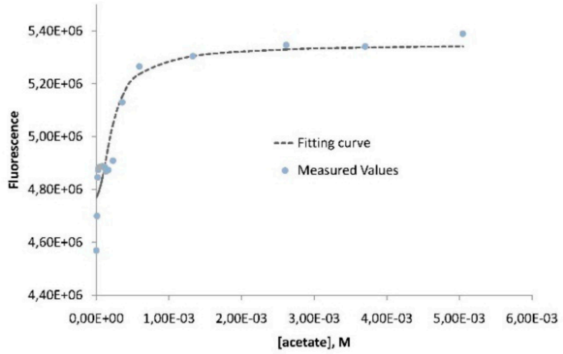

Cl^-

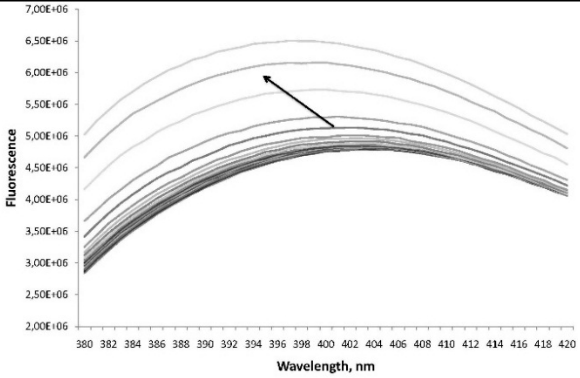

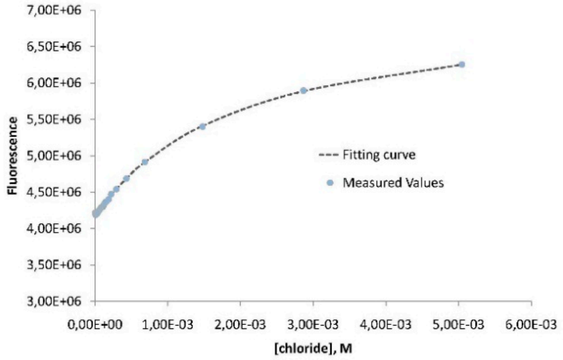

Table S3. Changes in UV-Vis spectra of receptor **2** upon addition of anions (**left**) and the data fitting (**right**).

$H_2PO_4^-$

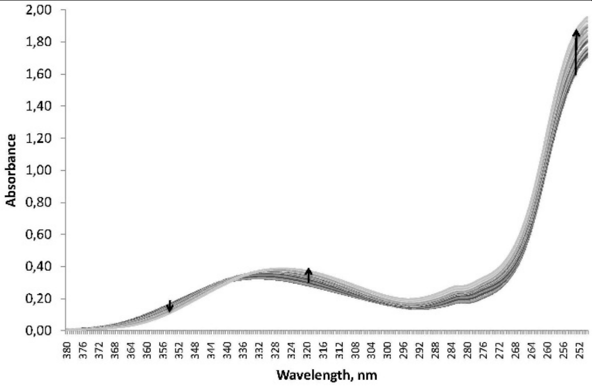

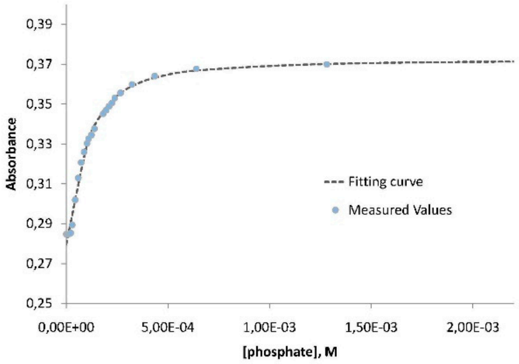

Table S3. *Cont.*

HSO_4^-

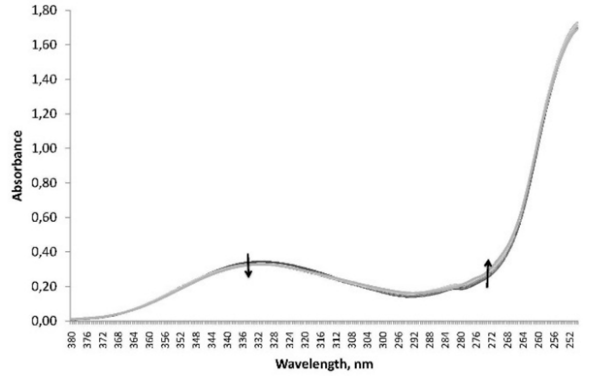

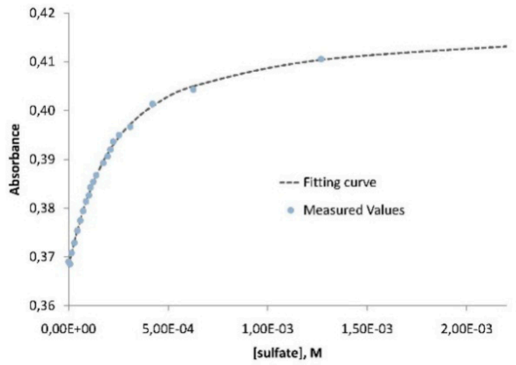

CH_3COO^-

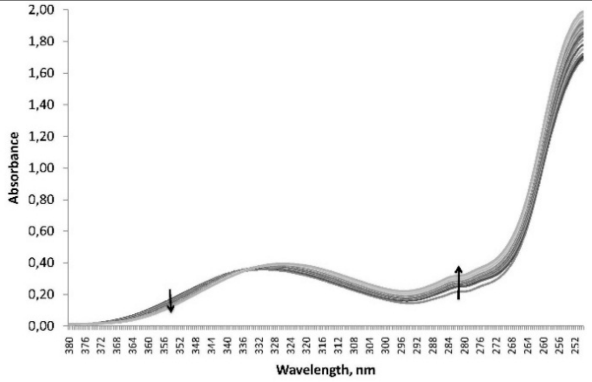

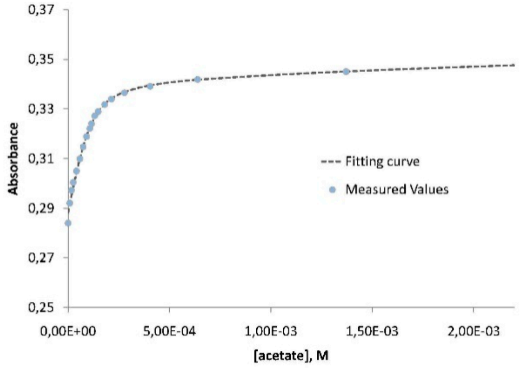

Cl^-

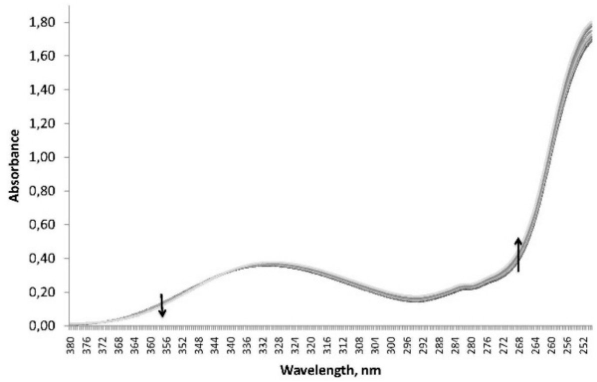

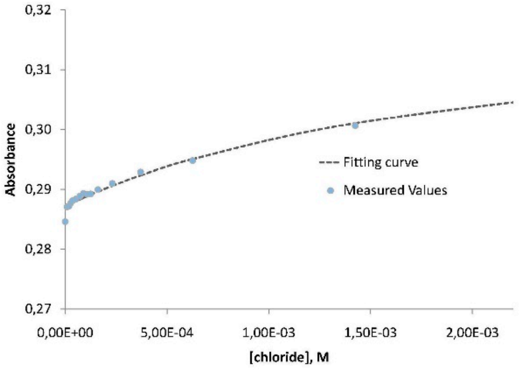

Table S4. Changes in fluorescence spectra of receptor **2** upon addition of anions (**left**) and the data fitting (**right**).

$H_2PO_4^-$

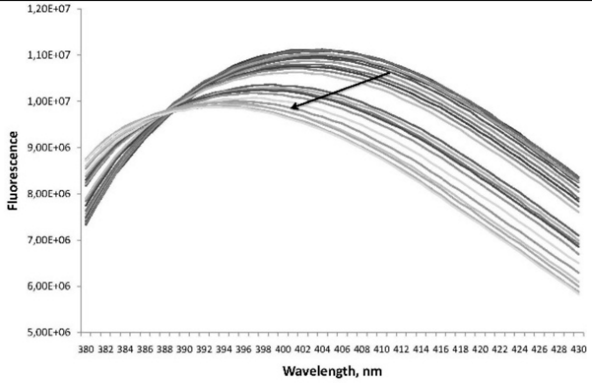

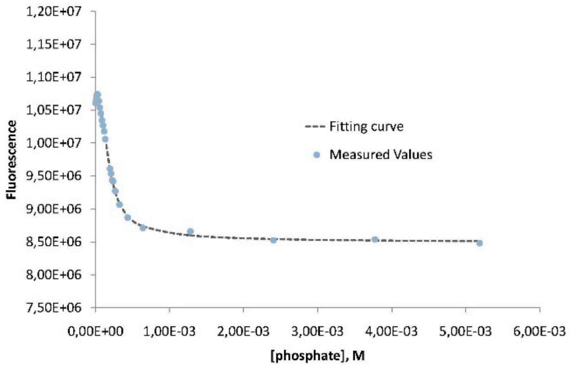

Table S4. *Cont.*

<table>
<tr><th colspan="2">CH_3COO^-</th></tr>
<tr><td>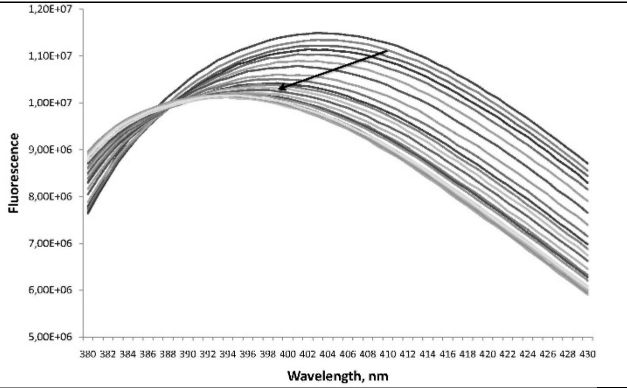
</td><td>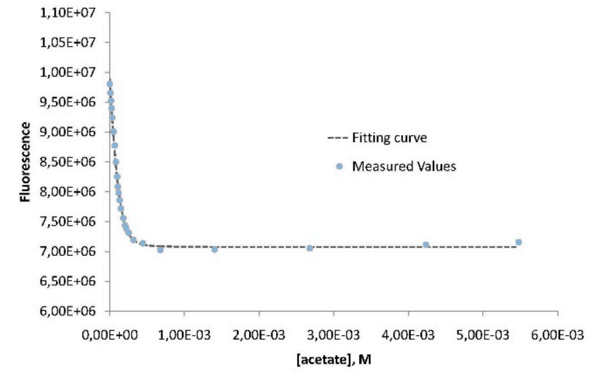
</td></tr>
<tr><th colspan="2">Cl^-</th></tr>
<tr><td>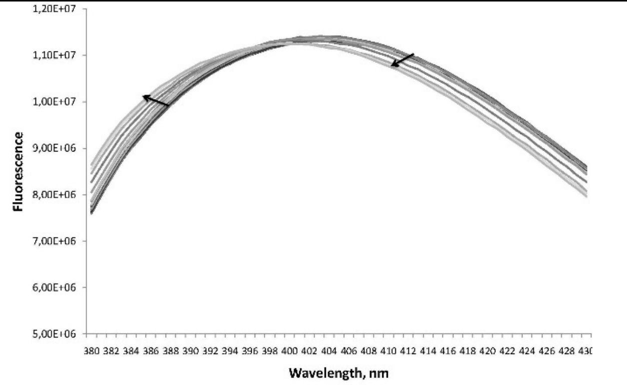
</td><td>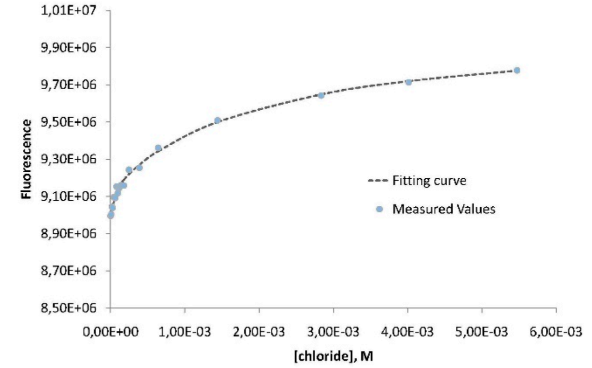
</td></tr>
</table>

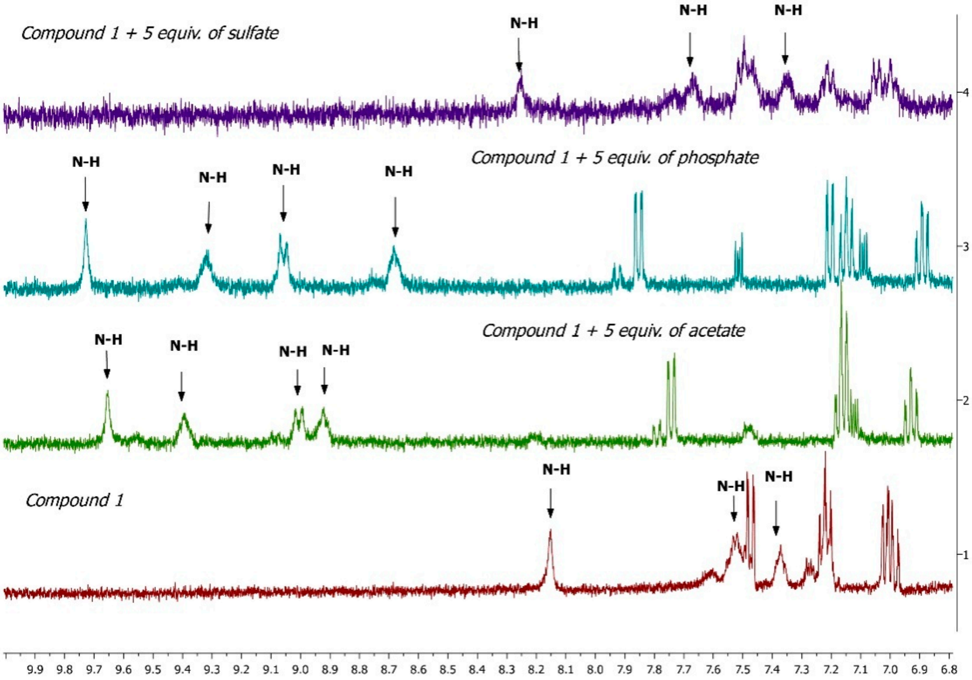

Figure S10. Comparison of the NMR spectrum of receptor **1** (1 mM in CD_3CN) with the spectra obtained after addition of five equivalents of anions.

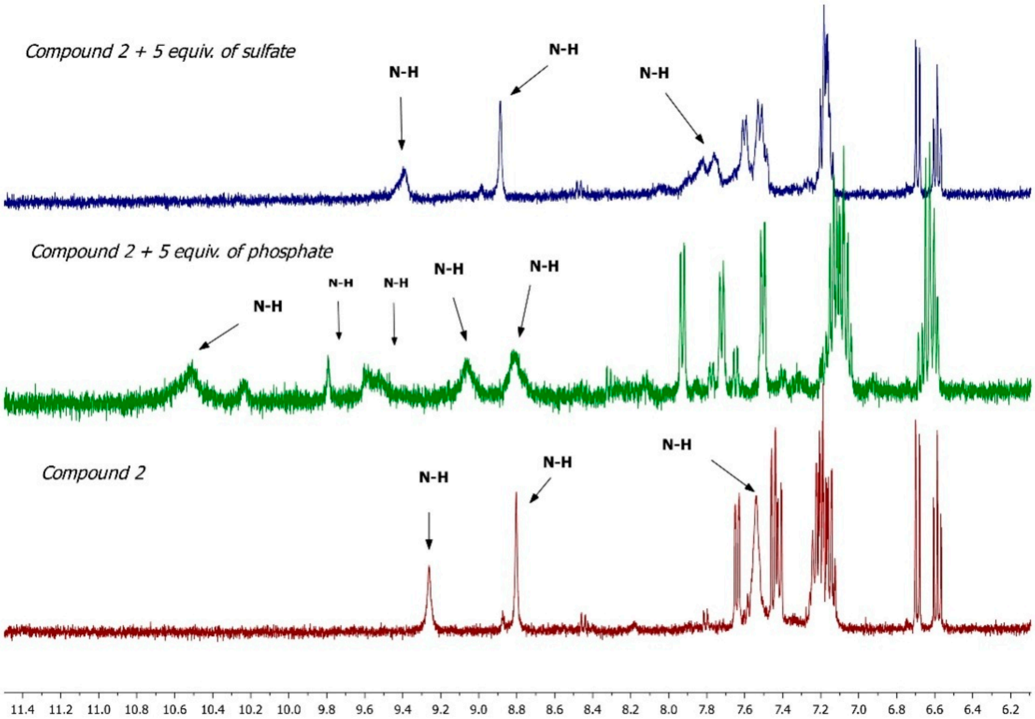

Figure S11. Comparison of the NMR spectrum of receptor **2** (1 mM in CD_3CN) with the spectra obtained after addition of five equivalents of anions. The spectrum of the mixture 1 + 5 equiv. of acetate is not shown because acetate induce a hydrolysis of the receptor at this concentration.

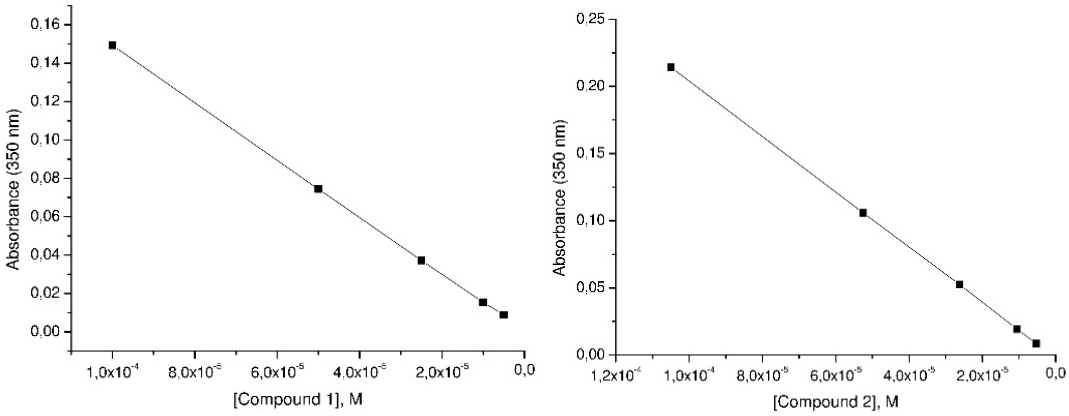

Figure S12. Absorbance-concentration dependence for receptor **1** (**left**) and **2** (**right**).

Table S5. Structures of the optimized host-guest complexes.

Coordinates of the Optimized Complex $1\bullet(H_2PO_4)_2$:	Coordinates of the Optimized Complex $2\bullet(H_2PO_4)_2$:
6 0.17012031 1.95183369 0.93084832	6 1.47231829 −3.81818104 0.07441166
6 0.75002805 −0.02289176 −0.34835704	6 3.00261855 −2.47808456 −1.30453765
7 1.03748179 0.93617567 0.69071934	7 2.54661179 −3.00278735 −0.01696694
6 −0.26003411 −1.11000794 0.08025722	6 3.48499084 −1.02227938 −1.18513215

Table S5. *Cont.*

6	−0.65019580	−1.86624484	−2.33612194	6	2.77790046	1.27438831	−0.63503832
7	−0.75025176	−1.94461555	−0.87514623	7	2.48838043	−0.11315435	−0.96635604
6	−1.50409466	−0.71624540	−2.96816603	6	2.40322137	2.29649162	−1.73429966
6	0.39627794	2.81921768	2.18516979	6	1.15268433	−4.33619118	1.50191927
7	1.67523047	2.74330252	2.89146297	7	0.68314338	−3.31596041	2.43207502
6	0.77827665	−1.89431612	−2.91062989	6	2.14118457	1.61240351	0.74115729
7	−2.78393683	−0.66622961	−2.47240791	7	1.23524845	2.03765678	−2.41227770
8	−1.05394742	−0.00269933	−3.87051262	8	3.10690594	3.29919672	−1.90237677
6	−3.85194804	0.12924010	−2.94370404	6	0.64689785	2.88704515	−3.37796712
6	3.65037953	3.73854225	3.95189150	6	−1.04190087	−1.81727457	3.33581734
6	4.69685342	4.61090703	3.60853817	6	−0.40689114	−1.67679477	4.58209276
6	5.89093757	4.65328877	4.32940511	6	−0.85085177	−0.74196863	5.52179480
6	6.03794046	3.82177197	5.44938397	6	−1.94265139	0.07890703	5.21618748
6	5.00769062	2.96007131	5.82015944	6	−2.59261370	−0.02819358	3.98559332
6	3.80606498	2.89273700	5.08091103	6	−2.15779352	−0.97415942	3.03330660
6	−5.18547471	−0.25592635	−2.60397849	6	−0.77149272	3.00066614	−3.44799781
6	−3.65035290	1.26682887	−3.74570393	6	1.43806481	3.63662004	−4.26624584
6	−4.73000768	2.01321114	−4.23176567	6	0.85309821	4.48834991	−5.20610619
6	−6.03806493	1.63207360	−3.91616388	6	−0.53996623	4.59605789	−5.27733421
6	−6.25308152	0.51271232	−3.10862213	6	−1.34373701	3.85467887	−4.40792656
7	−5.44491533	−1.32646398	−1.74868085	7	−1.57230318	2.25719118	−2.54675078
7	2.82236353	1.97807905	5.48692202	7	−2.75918841	−1.10190248	1.77499044
1	1.81009136	0.70468152	1.36391942	1	3.15661955	−2.88506293	0.81848997
1	−1.57504824	−2.50731048	−0.57937840	1	1.50330424	−0.40216747	−1.04159629
8	3.33651919	−0.83607361	4.76689619	1	3.74679852	−0.75395441	4.71074724
15	2.71012316	−0.97041245	3.24407220	1	5.75422192	−3.84922171	3.88956332
8	3.18270018	−2.23769452	2.57850963	8	2.61704206	−2.88554072	4.38781786
8	2.97101056	0.40345574	2.53829569	15	3.98663568	−2.65643191	3.74286509
8	1.09339040	−0.99758105	3.62664320	8	5.10605335	−3.58247256	4.56265783
1	2.20267635	1.81948295	2.89386477	8	4.45782185	−1.11086190	4.15349150
1	−2.99436491	−1.36342176	−1.72875372	8	4.23082924	−2.88217092	2.25642967
1	4.53032970	5.27849524	2.76163101	1	1.35143590	−3.02673316	3.17813945
1	6.69441424	5.32778137	4.02778929	1	0.73919749	1.14119816	−2.20889926
1	6.96136016	3.83772773	6.03329507	1	0.46247968	−2.29210496	4.82018185
1	5.13001390	2.29690341	6.68050846	1	−0.33702806	−0.65374428	6.48085642
1	−2.62852009	1.54686758	−3.98611012	1	−2.29523206	0.81816250	5.93981409
1	−4.53870681	2.89397790	−4.84750112	1	−3.43411732	0.61013633	3.72690463
1	−6.89176446	2.20382838	−4.28749446	1	2.51896763	3.54806256	−4.19239807
1	−7.27166070	0.21607550	−2.84207032	1	1.49107134	5.06044436	−5.88348627
1	−6.38100571	−1.71277434	−1.82281030	1	−1.00992453	5.25374746	−6.01218224
1	2.72272813	1.87949432	6.49363791	1	−2.42629933	3.94146776	−4.44208860
8	−0.60556292	−1.25450814	1.27065921	1	−1.18756461	1.33589590	−2.24783945

Table S5. *Cont.*

1	1.42762781	−2.43182972	−2.19993328	6	−3.74112248	−0.33880836	1.18758512
6	0.87720705	−2.57513417	−4.29495734	1	−2.36776710	−1.92172635	1.26248991
6	2.21585712	−2.22263342	−4.96333982	8	4.68369055	−0.73004937	−1.26090205
6	0.70869598	−4.10119879	−4.20229649	1	2.50253344	0.82253093	1.41916895
1	2.30580319	−2.69663126	−5.95434718	6	2.47933316	2.99566317	1.35033095
1	2.31451788	−1.13480870	−5.09476795	6	1.49610448	4.09755564	0.91946930
1	3.06297190	−2.56624300	−4.34641157	6	2.51891971	2.88882399	2.88616371
1	0.72888294	−4.56171136	−5.20318574	1	1.76596069	5.06212997	1.38050497
1	1.52391940	−4.54582625	−3.60791166	1	1.49345076	4.24330187	−0.16815652
1	−0.24077089	−4.38816847	−3.72626108	1	0.47128662	3.84623623	1.23947465
1	0.06883844	−2.16791703	−4.92587794	1	2.76133823	3.86052585	3.34720707
1	1.15384629	−0.86525530	−3.00052385	1	1.54079735	2.56456733	3.27893829
1	1.68916583	−0.52442379	−0.62574557	1	3.27071667	2.15470171	3.21363616
1	0.35447466	0.49688695	−1.23419045	1	3.48714519	3.28154731	1.00146973
8	−0.81686759	2.20186413	0.22636998	1	1.04582572	1.48991179	0.67700720
1	0.23098225	3.86313536	1.89038593	1	2.17279816	−2.55419683	−2.02565312
6	2.35798346	3.89125641	3.17335054	1	3.85756040	−3.06031752	−1.68071580
8	1.96859575	5.03403820	2.87421993	8	0.77995425	−4.17481327	−0.89672577
1	−1.16349245	−2.78649233	−2.66497429	1	0.38569248	−5.11079550	1.37169158
1	−0.42721075	2.53642518	2.86449623	6	−0.58131856	−2.84638453	2.33288574
1	1.92783618	2.07077829	5.00294507	8	−1.35334754	−3.25471020	1.43107128
1	−4.71930108	−2.05747370	−1.61128529	6	−3.92405605	−0.64123583	−0.31236237
8	−3.33101396	−2.87316392	−0.73491597	7	−3.12160587	0.25207645	−1.16727364
15	−3.90107730	−3.17623115	0.70226943	6	−3.47508526	1.67224765	−1.06755650
8	−2.66060882	−3.21082494	1.76453778	6	−2.81184745	2.62178922	−2.08224511
8	−4.76406005	−4.39873894	0.88355655	8	−4.37996292	0.56289107	1.74782097
8	−4.68797882	−1.77582891	1.15639740	1	−3.62567830	−1.67335713	−0.55084389
1	−2.01604494	−2.46776787	1.64605275	1	−4.98936319	−0.50222760	−0.55289268
1	0.53983516	−1.11410049	2.81880704	8	−3.39536572	3.67421699	−2.36810279
1	3.25818918	0.11236162	5.02458347	1	3.87135148	1.34442627	−0.54957402
1	−5.02227734	−1.34106779	0.34354331	1	−3.27785301	2.10639644	−0.06642279
				1	−4.55681372	1.77865553	−1.23423922
				1	−2.11743355	0.09700878	−0.97164416
				1	2.06480622	−4.76804543	1.93889010
				1	−2.65575838	−0.50301617	−2.91716075
				8	−1.26883137	−2.60431504	−1.91172838
				15	−0.67557126	−1.36946428	−2.81894946
				8	−0.34073564	−0.19015333	−1.81491399
				8	−2.05531669	−0.91721737	−3.59308195
				8	0.40637699	−1.78266299	−3.78423882
				1	−0.55011326	−3.18189573	−1.54733181
